# Sequential Galacto- and Xylo-Oligosaccharide Feeding Transiently Modulates Gut Microbiota and Upregulates Intestinal Alkaline Phosphatase in Weaning Piglets

**DOI:** 10.3390/ani15213210

**Published:** 2025-11-04

**Authors:** James S. Stanley, Stephen C. Mansbridge, Michael R. Bedford, Ian F. Connerton, Kenneth H. Mellits

**Affiliations:** 1 Division of Microbiology, Brewing, and Biotechnology, School of Biosciences, University of Nottingham, Loughborough LE12 5RD, UK; ian.connerton@nottingham.ac.uk; 2 Animal Science Research Centre, Harper Adams University, Newport TF10 8NB, UK; smansbridge@harper-adams.ac.uk; 3 SBNS Ltd., Wiltshire SN8 1QJ, UK; mikebedf@googlemail.com

**Keywords:** prebiotics, oligosaccharides, dietary carbohydrates, pigs, growth, microbiota, intestinal health, histology, immunity

## Abstract

**Simple Summary:**

Neonatal piglets reared for human consumption are weaned from the sow at an early age, well before their gastrointestinal tract has fully developed. During weaning, piglets must adapt to a sudden dietary shift from highly digestible, liquid sow’s milk to a more complex, solid, cereal-based feed. The abrupt diet change, combined with other stressors and an immature gut, disrupts the gut microbiota, predisposing piglets to post-weaning diarrhoea and growth checks. These cause both economic loss for producers and welfare concerns for the animals. Historically, antibiotics were added to livestock feeds to reduce post-weaning diarrhoea and improve feed efficiency. However, the use of antibiotics as growth promoters has been banned in many territories, creating a need for alternative strategies to support piglet health during weaning. In this study, we tested whether two prebiotic dietary fibres could ease the weaning transition by stimulating beneficial bacteria in the gut, with the aim of improving gut health and growth. Our results show that these prebiotics briefly altered the gut microbiota and enhanced markers of gut health but did not improve feed efficiency or growth.

**Abstract:**

Improving growth and health at weaning remains a priority in pig production. This study investigates whether supplementation with galacto-oligosaccharides (GOSs) followed by xylo-oligosaccharides (XOSs) improves performance and gut health of healthy 28-day old weaning piglets. Pigs received either a control basal (CON) diet, the CON diet containing 1% GOS for 7 days followed by the CON diet containing 0.017% XOS for 47 days (GXOS), or the CON diet for 7 days followed by the CON diet containing 0.017% XOS for 47 days (XOS). Body weight, average daily gain, average daily feed intake, and feed conversion ratio did not differ between diets from day 1 of weaning (d1) to d54. At d7, GXOS pigs showed increased jejunal and caecal α-diversity (Shannon, inverse Simpson), distinct ileal β-diversity (Yu and Clayton, Bray–Curtis, Jaccard), and greater short-chain fatty acid-producing *Lactobacillus* and *Veillonella*; no taxa remained differentially abundant by d22, and the XOS group showed no microbiota shifts throughout the study. Jejunal goblet cell density was lower in GXOS pigs at d7. Jejunal and caecal *IL-1β*, *IL-6*, *IL-8*, and *IL-10* gene expression was transiently greater at d7 in GXOS pigs, whereas by d22 cytokine/chemokine differences resolved, whilst intestinal alkaline phosphatase was upregulated in the ileum and caecum (XOS) and colon (GXOS and XOS). Sequential prebiotic switching and delayed XOS onset likely missed the immediate post-weaning window, during which the gut microbiota is most receptive to dietary modulation, consequently limiting potential performance gains; therefore, prebiotic timing, sequence, and duration are critical to achieving functional benefits at weaning.

## 1. Introduction

Global demand for animal protein continues to rise, with total meat production projected to reach approximately 388 million metric tonnes by 2033, driven by population growth [1]. Pork remains one of the most consumed meats worldwide and the leading source of animal protein in Europe, placing pressure on producers to improve feed efficiency and sustainability [1]. In this regard, improving animal growth performance and nutrient utilisation is key to sustaining both economic viability and production efficiency.

Although gains in growth rate and feed efficiency have been achieved through selective breeding [2], the early-life period, particularly weaning, remains a major challenge for pig health and performance. In commercial production systems, piglets are typically weaned at 3–4 weeks of age, well before full gastrointestinal development and intestinal maturation [3]. This early separation from the sow deprives piglets of continued access to sow’s milk, which contains bioactive components such as maternal antibodies and oligosaccharides that confer passive immunity and support the establishment of a beneficial gut microbiota, contributing to the development of mucosal immunity and colonisation resistance [4,5]. The commercial weaning process also introduces multiple environmental and nutritional stressors whereby piglets must adapt to abrupt maternal separation, housing relocation, social mixing with other litters, and a sudden dietary shift from highly digestible, liquid milk to a more complex, cereal-based feed [6]. This abrupt change in nutrient substrate availability disrupts the milk-adapted microbial community and introduces novel polysaccharides that select for different degraders, resulting in community restructuring and dysbiosis [7]. These changes often lead to a transient reduction in voluntary feed intake, villus atrophy, reduced digestive enzyme activity, microbial dysbiosis, and gut inflammation, triggering a post-weaning growth check (PWGC) [6,7,8]. Early-life dysbiosis disrupts the normal co-development of the gut microbiota and immune system, reducing short-chain fatty acid (SCFA) production—particularly butyrate—which is critical for maintaining epithelial integrity and promoting T cell (Treg) differentiation via IL-10 and TGF-β signalling [9,10]. The resulting loss of SCFA-mediated immune tolerance, combined with increased luminal antigen exposure due to barrier dysfunction, enhances activation of pro-inflammatory pathways such as NF-κB and JAK–STAT, elevating *IL-1β*, *IL-6*, and *TNF-α* expression and further compromising barrier function [8,11,12,13]. Consequently, the immature and destabilised gut is more vulnerable to colonisation by enteric pathogens such as *Salmonella* spp. and enterotoxigenic *Escherichia coli* [14], which can compromise piglet health and welfare and contribute to increased morbidity and mortality during the post-weaning period, leading to substantial economic loss for the pig production industry [7]. It has been reported that earlier weaning hampers growth rate and market performance; piglets weaned at 19 days of age are approximately 18 kg lighter at slaughter when slaughter day is fixed, and require additional time to reach the same target weight as 28-day weaned piglets [15]. Such early-life growth inhibition carries direct economic implications: based on 2024 UK carcass prices (208 p kg^−1^; [16]), this weight difference equates to an estimated £37 lower revenue per pig. This underscores the substantial production and financial losses associated with post-weaning stress and highlights the need for nutritional strategies that stabilise gut function and promote growth.

Alongside advances in selective breeding, in-feed antimicrobial growth promoters (AGPs) have historically enhanced growth performance and feed efficiency in pigs [17]. However, their use was banned in the European Union in 2006 due to antimicrobial resistance concerns and the transfer of resistance genes from livestock to human microbiota, as well as evidence linking AGP use to reduced intestinal microbial diversity [18,19]. Similar restrictions have since been adopted in other territories worldwide, with China banning all antibiotic growth-promoting feed additives in 2020 and the United States eliminating growth-promotion uses of antimicrobials considered medically important for human medicine in 2017 [20,21].

The gastrointestinal tract (GIT) microbiota plays a key role in supporting host growth, health and welfare by contributing to nutrient metabolism, immune development, and gut barrier function [22]. Furthermore, early-life microbial colonisation and gut immune maturation exert lasting effects on the composition and activity of the adult intestinal microbiota and innate immune system, reflecting a developmental continuum from weaning through to maturity [23]. Moreover, the GIT microbiota encodes metabolic capabilities that are absent in the host genome. This is no more evident when considering the metabolism of plant cell wall polysaccharides that are a major constituent of post-wean pig diets. These complex carbohydrates are processed in the large intestine, where Bacteroidota species, possessing a diverse array of polysaccharide-degrading enzymes, anaerobically ferment them into bioavailable metabolites [24,25]. This microbial fermentation yields SCFAs, such as acetate, propionate, and butyrate, which serve as energy sources for colonocytes, modulate local and systemic inflammation, and lower intestinal pH, creating an environment less favourable for the proliferation of enteric pathogens [26,27]. Beneficial genera such as *Lactobacillus* and *Bifidobacterium* are well recognised for their SCFA synthesis and ability to outcompete pathogenic species through acidification of the gut lumen and competitive exclusion [27,28,29]. As such, maintaining a balanced and functionally active microbiota is essential not only for digestive efficiency but also for mitigating inflammation and supporting disease resilience.

Various feed supplements and additives, including prebiotics, probiotics, essential oils, organic acids, herbs, and exogenous enzymes, have been proposed as AGP alternatives to promote beneficial GIT microbiota [30]. The original concept of prebiotics was first introduced by Gibson et al. (1995) [30] and has since been generalised to “a substrate that is selectively utilised by host microorganisms conferring a health benefit” [31]. Prebiotic effects include modulation of microbiota composition, enhancement of intestinal barrier function, regulation of immune responses, and suppression of pathogens [32].

Galacto-oligosaccharides (GOSs) are non-digestible carbohydrates derived from lactose through enzymatic transgalactosylation, while xylo-oligosaccharides (XOSs) are produced from the hydrolysis of xylan, a plant-derived polysaccharide [33,34]. Dietary GOS has been shown to improve piglet growth performance, reduce intestinal inflammation, and modulate the gut microbiota by enriching beneficial bacteria and restricting opportunistic pathogens [35,36,37,38,39]. Additionally, GOS has been shown to alleviate *Salmonella enterica* serotype Typhimurium and *Escherichia coli* lipopolysaccharide-induced intestinal injury in pigs by modulating the gut microbiota and suppressing inflammatory cytokines [40,41]. Dietary intervention with XOS has been shown to enhance performance, reduce intestinal inflammation, and modulate the gut microbiota in healthy weaning piglets [42,43,44,45], as well as improve health to pigs in challenge models [46,47]. Furthermore, Stanley et al. (2025) [48] demonstrated that in piglets experiencing environmentally acquired post-weaning diarrhoea, XOS supplementation increases the abundance of SCFA-producing bacteria, enhances innate immunity, reinforces mucosal barrier integrity, and improves post-weaning growth, confirming its efficacy under conditions encountered in commercial production.

In weaning piglet studies, the standard prebiotic intervention typically involves supplementing animals with a single oligosaccharide for a defined period immediately post-weaning [49]. However, this approach overlooks the weaning transition from sow’s milk—rich in GOS—to cereal-based feeds, which are abundant in xylan, the hemicellulose polymer from which XOS are derived [50,51]. Commercially synthesised GOS, although structurally simpler than milk oligosaccharides (being predominantly β-(1→4)-linked galactose units, whereas porcine milk oligosaccharides are mainly 3′- and 6′-sialyllactose [34,52]), shares functional similarities by selectively stimulating *Lactobacillus* and *Bifidobacterium*, and by promoting mucosal development and cross-feeding networks that support microbial stability and intestinal health during the critical post-weaning period [35,36,37,38,39]. Early exposure to GOS may therefore help stabilise the weaning microbiota and promote beneficial taxa capable of fermenting more complex carbohydrates introduced later in the diet. Subsequent supplementation with XOS could sustain these networks by providing substrates better matched to the maturing, cereal-adapted gut environment. On this basis, as nutritional support for piglets undergoing the dietary transition at wean, we have developed a sequential supplementation strategy whereby an initial 7-day supplementation of GOS immediately post-weaning, is followed by 47 days of XOS supplementation, aimed to mitigate post-weaning gut microbiota dysbiosis and the associated PWGC. Therefore, the objectives of this study were to investigate the prebiotic effects of GOS microbiota pre-conditioning before extended XOS supplementation on growth performance, gut microbiome composition, immune response, gut barrier integrity, intestinal architecture, and goblet cell (GC) expression in weaning piglets. The prebiotic doses used in the present study were selected based on previous reports that demonstrate significant effects on growth performance in piglets supplemented with GOS [37,38] or XOS [42,53,54].

## 2. Materials and Methods

### 2.1. Ethical Approval

All experimental procedures were approved by the Harper Adams University Research Ethics Committee (Approval Reference Number: 0063-202401-STAFF) and the University of Nottingham Animal Welfare and Ethical Review Body (Approval Reference Number: 40). Animals were humanely killed in accordance with the requirements of the Animals (Scientific Procedures) Act 1986 (as amended). This study and manuscript comply with the ARRIVE 2.0 guidelines [55].

### 2.2. Experimental Animals and Trial Design

A total of 216 (108 male, 108 female) healthy piglets (JSR 9T dam × JSR Tempo sire cross), averaging 8.32 ± 0.04 kg body weight at weaning, were used to investigate the effects of in-feed GOS and XOS on growth performance and gut health compared to a basal diet with no prebiotic supplementation. On day 1 of weaning (d1), piglets were randomly assigned to one of three dietary groups: (1) a control basal (CON) diet; (2) the CON diet supplemented with 1% GOS (*w*/*w* GOS/feed) for seven days followed by the CON diet supplemented with 0.017% XOS (*w*/*w* XOS/feed) for 47 days (GXOS); or (3) the CON diet for seven days followed by the CON diet supplemented with 0.017% XOS (*w*/*w* XOS/feed) for 47 days (XOS). Each diet group comprised 12 replicate pens containing six pigs per pen (balanced across sex; three males and three females per pen), with pig and diet assignment to pens performed using a random number generator. Sample size was calculated a priori using Berndtson’s procedure [56], with assumptions of a 10% detectable difference in body weight, 5–8% coefficient of variation, 80% statistical power, and α = 0.05, in line with EFSA FEEDAP (2018) recommendations [57]. The trial lasted 54 days, with feed and water provided ad libitum. The formulation and nutrient composition of the basal diets used throughout the study are provided in Table 1. Non-edible chew toys and chains were provided as environmental enrichment while preventing potential microbiome interference. The study was conducted at the Harper Adams University Future Farm Pig Unit, Shropshire, UK. Neonates were reared in farrowing pens (4.3 m^2^, temperature-controlled) with a sow until weaning at 28 days of age (da), after which pigs were transferred to group pens (2.45 m^2^, plastic slatted floors). Stocking density was 0.41 m^2^ per pig from weaning to d7; 0.49 m^2^ per pig from d8-d22; and 0.61 m^2^ per pig from d23-d54. The nursery temperature was gradually reduced following a controlled temperature curve from 28 °C on d1 to 20 °C by d54, and a light/dark cycle was maintained from 08:00 h to 16:00 h. Relative humidity and ventilation of the nursery were set in accordance with the Red Tractor Assurance for Farms Pig Scheme Standards at the time of the study. At weaning, pigs were vaccinated with Porcilis PCV M Hyo (Intervet International BV, MSD Animal Health, Boxmeer, The Netherlands; VM:EU/2/08/091/001-010) for active immunisation against Porcine circovirus type 2 and *Mycoplasma hyopneumoniae*. Prebiotics were supplied as dry powders: GOS with degree of polymerisation >2 (DP2+) were obtained from Saputo Dairy UK (Weybridge, UK) with 70% purity and XOS with DP2+ were obtained from Shandong Longlive Bio-technology Co., Ltd. (Dezhou, Shandong, China) with 37.3% purity. Feeding phases comprised: starter (d1–d7), link (d8–d22), and grower (d23–d54). The prebiotics were incorporated by dry mixing into the formulated diets at their specified inclusion rates. Mixed diets were then pelleted at a commercial mill to produces batches of 125 kg creep feed, 225 kg starter feed, 950 kg link feed, and 3825 kg grower feed (per treatment, per diet stage; Primary Diets Ltd., Ripon, UK). Wheat-soya-based diets met or exceeded all nutrient requirements of BSAS 2003 [58] and contained no antibiotics, growth promoters, or probiotics; formulations are provided in Table S4. At birth, piglets were tagged with electronic ear tags for identification and housed in litters with continuous access to a sow and offered proprietary supplementary milk (VIDA Milk, Bury St Edmunds, UK) until weaning. Piglets were offered proprietary creep feed (Progress 2, Primary Diets Ltd., Ripon, UK) from 15 da to weaning. The nutritional compositions of supplementary milk and creep feed are provided in Tables S5 and S6. Sows received Porcilis^®^ Porcoli Diluvac Forte (Intervet International BV; Vm:EU/2/96/001/003-008) three weeks before farrowing for passive protection against neonatal enterotoxigenic *Escherichia coli* causes of enterotoxicosis. Animals were not subject to regulated procedures that may cause pain, suffering, distress, or lasting harm exceeding the lower threshold, as defined under the UK Animals (Scientific Procedures) Act 1986 (as amended). Animals were monitored daily for signs of ill health (e.g., failure to gain weight, diarrhoea, respiratory distress, and lameness), with predefined humane endpoints set to include sustained systemic illness, weight loss, or severe lameness, at which point animals would be removed from the study and treated or euthanised in consultation with the named animal welfare officer or named veterinary surgeon. All pigs were weighed on days 1, 7, 14, 22, and 54 post-weaning to monitor production performance, and pen-level feed intake was monitored to calculate average daily gain (ADG), average daily feed intake (ADFI), and feed conversion ratio (FCR). For each pen, ADG, ADFI, and FCR were calculated as follows: ADG = total weight gain/total study days; ADFI = total feed intake/total study days; and FCR = total feed intake/total weight gain. The ADG, ADFI, and FCR were calculated for the entire experimental period (d0–d54) to capture cumulative effects of the sequential feeding strategy, rather than phase-specific responses. On d7 and d22, one pig per pen (closest to the pen’s mean weight) was euthanised by penetrative captive bolt and pithing for sample collection. Investigators were unblinded for farm work to ensure correct feed allocation but blinded during laboratory and statistical analyses.

### 2.3. Sample Collection

At each sampling day (days 7 and 22 post-weaning), one pig per pen (*n* = 12 pigs per diet group per timepoint) was humanely euthanised for intestinal sampling. Upon death, the entire GIT was excised from each animal, and intestinal sections were identified and secured with zip ties to prevent the flow of luminal contents into adjacent regions. At least 10 g (up to the maximum volume that could be collected within a 70 mL container) of luminal digesta was aseptically collected from the midpoints of the duodenum, jejunum, and ileum; the apical tips of the caecum and colon; and the most distal portion of the rectum. Luminal contents were snap frozen on dry ice, transferred to the laboratory, and stored at −80 °C until DNA isolation for microbiota analysis. A 1 cm thick cross-section was excised post-mortem from the midpoints of the jejunum and ileum and preserved in 10% neutral buffered formalin (NBF) specimen containers (Fisher Scientific, Loughborough, UK) for histological analysis and goblet cell enumeration. Approximately 1 cm^2^ of intestinal tissue was excised from the midpoints of the duodenum, jejunum, and ileum, as well as from the apex of the caecum and colon, and preserved in cryotubes containing 1.5 mL RNAlater RNA Stabilisation Reagent (Invitrogen, Thermo Fisher Scientific, Loughborough, UK), snap frozen in liquid nitrogen, and transferred to the laboratory and stored at −80 °C until RNA isolation for gene expression analysis.

### 2.4. DNA Isolation and PCR Amplification of 16S rRNA Gene Sequences

Bacterial DNA was isolated from 200 mg of luminal contents using the QIAamp 96 PowerFecal QIAcube HT Kit and the QIAcube HT (Qiagen, Hilden, Germany) according to the manufacturer’s instructions, with minor modifications: weighed digesta was homogenised using Pathogen Lysis Tubes S (Qiagen, Hilden, Germany) on the FastPrep-24 5G bead-beater system (MP Biomedicals, Solon, OH, USA). For each sample, the V4 region of the bacterial 16S rRNA genes were polymerase chain reaction (PCR) amplified using the primers 515f (5′ GTGCCAGCMGCCGCGGTAA 3′) and 806r (5′ GGACTACHVGGGTWTCTAAT 3′) [59]. The PCR cycling conditions were initial enzyme activation at 95 °C for 2 min, followed by 30 cycles of denaturation at 95 °C for 20 s, annealing at 55 °C for 15 s, and extension at 72 °C for 5 min, with a final extension at 72 °C for 10 min. The PCR products were cleaned, normalised, and pooled using the SequalPrep Normalization Plate Kit (Thermo Fisher Scientific, Loughborough, UK). The DNA library was assessed for fragment size distribution using the Agilent D1000 reagent and ScreenTape kits with the Agilent 2200 TapeStation System (Agilent Technologies, Inc., Santa Clara, CA, USA). The DNA library concentration was assessed using the Qubit dsDNA HS Assay kit with the Qubit 4 Fluorometer (Thermo Fisher Scientific, Loughborough, UK). Libraries were sequenced on the Illumina MiSeq platform (Illumina, Cambridge, UK) using 2 × 250 bp cycles. Sequence data were deposited in the NCBI database within the BioProject PRJNA1308109, with SRA records available at https://www.ncbi.nlm.nih.gov/sra/PRJNA1308109 (accessed on 20 August 2025).

### 2.5. Microbiota Diversity Analysis

The 16s rRNA gene sequences were quality filtered and clustered into operational taxonomic units (OTUs) using Mothur v.1.48.1, following the MiSeq SOP (https://www.mothur.org/wiki/MiSeq_SOP, accessed on 25 June 2025) [60,61]. Reads were aligned to the SILVA bacterial reference alignment (v.138.2; available at https://mothur.org/wiki/silva_reference_files, accessed on 25 June 2025) [62] and clustered into OTUs using the “OptiClust” algorithm at 3% cut-off [63]. The consensus taxonomy for each OTU was assigned using the “classify.otu” command in Mothur, with reference data from the Mothur-formatted Ribosomal Database Project (v.19) [64,65]. Rarefaction curves were generated using resampling without replacement to assess sampling effort. Command syntaxes for use in Mothur are deposited at https://github.com/J-S-Stanley/GOS-XOS-2025 (accessed on 19 September 2025).

### 2.6. RNA Isolation, RT-qPCR, and Gene Expression Analysis

To assess host gene expression, RNA was isolated from duodenal, jejunal, ileal, caecal, and colonic tissue. For each sample, 15 mg tissue was homogenised in 600 µL Buffer RLT (Qiagen, Hilden, Germany) containing 1% (*v*/*v*) β-mercaptoethanol using 2 mL nuclease-free Lysing Matrix D tubes (MP Biomedicals, Solon, OH, USA) on the FastPrep-24 5G bead-beater system (MP Biomedicals, Solon, OH, USA). Total RNA was purified with the QIAamp RNeasy 96 QIAcube HT Kit and the QIAcube HT (Qiagen, Hilden, Germany), according to the manufacturer’s instructions, eluted in nuclease-free water, quantified and quality-checked using UV spectrophotometry (Biochrom BioDrop Duo+; Biochrom, Harvard Bioscience, Inc., Holliston, MA, USA), and stored at −80 °C until reverse transcription quantitative PCR (RT-qPCR) within 1 week. Residual genomic DNA was removed, and RNA was reverse transcribed to cDNA using the RT^2^ First Strand Kit according to the manufacturer’s instructions (Qiagen, Hilden, Germany). Each 10 µL reverse transcription reaction contained 4 µL buffer BC3, 1 µL Control P2, 2 µL RE3 Reverse Transcriptase Mix, and 3 µL nuclease-free water. Reactions were incubated at 42 °C for 15 min for reverse transcription, followed by enzyme inactivation at 95 °C for 5 min. Following thermal cycling, 91 µL nuclease-free water was added to each cDNA reaction. Custom RT^2^ Profiler PCR Assays (Qiagen, Hilden, Germany) with pre-attached primer sets were used to quantify transcripts encoding tight junction proteins *TJP1* (zonula occludens-1; ZO-1), *TJP2* (zonula occludens-2; ZO-2), *OCLN* (occludin), *CLDN2* (claudin-2), and *CLDN-3* (claudin-3); cytokines *IL-10* (interleukin-10), *IL-6* (interleukin-6), and *IL-1*β (interleukin-1β); the chemokine *IL-8* (interleukin-8); and the brush border enzyme *ALPI* (intestinal alkaline phosphatase). Ribosomal protein L4 (*RPL4*) and glyceraldehyde-3-phosphate dehydrogenase (*GAPDH*) were used as reference genes (HKGs). Assay IDs for target and reference genes are provided (Table S7). Target gene expression was determined using qPCR with the RT^2^ SYBR Green qPCR Mastermix kit (Qiagen, Hilden, Germany) on the Roche Diagnostics LightCycler 480 Instrument (Hoffman La Roche, Basel, Switzerland). Each 10 µL qPCR reaction contained 1 µL cDNA, 4 µL nuclease-free water, and 5 µL SYBR Green qPCR Mastermix. The qPCR cycling conditions were enzyme activation at 95 °C for 10 min, followed by 45 cycles of denaturation at 95 °C for 15 s and combined annealing/extension at 60 °C for 1 min with single data acquisition. A post-amplification melt-curve was performed from 60 °C to 95 °C (0.03 °C s^−1^ ramp rate, 20 acquisitions °C^−1^) to verify product specificity. Relative target gene expression was determined using the 2^−ΔΔct^ method, with ct as the qPCR cycle threshold, Δct as the difference between the target gene ct and the average HKG ct, and ΔΔct as the difference in transcript levels between CON and prebiotic groups. Statistical analysis was performed on the 2^−ΔΔct^ values.

### 2.7. Histology Analysis

Formalin-fixed tissues were processed using an Epredia™ Excelsior™ AS tissue processor (Epredia Holdings Ltd., Portsmouth, NH, USA) based on previous methods [48]. Briefly, samples underwent graded alcohol dehydration, were cleared using xylene, and were embedded in paraffin wax. Sections were cut at 5 µm thickness using a microtome, mounted on glass slides, and stained using the periodic acid–Schiff (PAS) method. PAS-stained slides were subsequently scanned using the Roche VENTANA DP^®^ 200 slide scanner at 40× magnification (Hoffman La Roche, Basel, Switzerland). Histological analysis was performed using QuPath software (v.0.5.1) [66]. For each scanned slide, all well-oriented villus–crypt pairs were identified; when ten or more pairs were present, ten were randomly selected for measurement using a random number generator, whereas all pairs were analysed when fewer than ten were available. This approach reflects common practices in porcine intestinal morphometric analysis [67,68]. Slides with inadequate staining or scan quality that precluded reliable identification/measurement of villi, crypts and GC density were removed from analysis. Villus height, crypt depth, villus area, and crypt area were measured, and goblet cells were enumerated in both villus and crypts within QuPath. Villus height was defined as the distance from the villus tip to the crypt opening, while crypt depth extended from the opening to the crypt base. The villus-to-crypt ratio (VCR) was calculated as villus height divided by crypt depth. For each pig, intestinal section, and metric (villus height, crypt depth, VCR, and GC density), measurements from villus–crypt pairs were averaged to obtain a single mean value per metric. The mean values were used for statistical analysis, with the individual pig considered the experimental unit.

### 2.8. Statistical Analyses

All statistical analyses and data visualisation were conducted in R (v.4.2.1) [69] using RStudio (v.2023.06.0+421) [70] unless otherwise specified. Data normality was evaluated using the Shapiro–Wilk test [71], and non-parametric or parametric methods were applied as appropriate. Multiple comparisons were adjusted for false discovery rate using the Benjamini–Hochberg procedure, following Tukey’s honest significant difference (HSD) or Dunn’s tests [72]. For all analyses, because the CON and XOS groups received identical basal diets during d1–d7, data from these groups were pooled, labelled, and presented as CON for analyses at d7. The group receiving GOS from d1–d7 followed by XOS from d8–d54 was designated as GXOS for both d7 and d22 for consistent labelling across timepoints, although only GOS was supplemented during d1–d7. From d8–d54, the three diets (CON, GXOS, XOS) differed and were analysed and presented as three separate groups. For performance metrics (BW, ADG, ADFI, FCR), the pen was considered the experimental unit, as pen averages were used for analysis. For microbiota, histology, and gene expression analyses, the individual pig was the experimental unit, since one pig per pen was euthanised at each sampling timepoint, and each sample represented one biological replicate. Body weights at d1, d7, d14, d22, and d54, and ADG, ADFI, and FCR were compared across diets using *t*-tests/one-way ANOVA or Wilcoxon rank sum/Kruskal–Wallis tests, as appropriate, with Tukey’s HSD or Dunn’s tests for pairwise comparisons. Samples containing a total number of sequences less than 18,963 sequences were excluded from microbiome analyses, ensuring sufficient sequencing depth and data quality. The “summary.single” command in Mothur was used to calculate Good’s coverage [73] and α-diversity indices (ACE Estimator [74], Chao richness [75] inverse Simpson diversity [76] and Shannon index [77]). Wilcoxon rank sum tests (d7) and Kruskal–Wallis with Dunn’s tests for post hoc comparisons (d22) were used to measure differences in α-diversity. The β-diversity indices (Jaccard similarity [78], Bray–Curtis dissimilarity [79], and Yue and Clayton dissimilarity [80]) were calculated and assessed for significance using analysis of molecular variance (AMOVA) in Mothur [81,82]. The relative abundance of bacterial taxa was calculated in R using Mothur output files. Differential abundance analysis (log_2_ fold change) of OTUs was performed using ANCOM-BC2 (v.2.0.3) in R on OTUs with ≥10% prevalence and >50 sequences [83]. Syntax for ANCOM-BC2 analysis in R is deposited at: https://github.com/J-S-Stanley/GOS-XOS-2025 (accessed on 19 September 2025). Spearman’s rank correlation was used to assess the relationship between the top four OTUs for each timepoint and intestinal site. Wilcoxon rank sum tests (d7) and Kruskal–Walis with Dunn’s tests for post hoc comparisons (d22) were used to assess differences in villus height, crypt depth, VCR, and GC density between diets. For gene expression analysis, values with ct >35 were excluded. Outliers were defined as ct below Q1 − (1.5 × IQR) or above Q3 + (1.5 × IQR) and removed prior to analysis. Wilcoxon rank sum tests (d7) and Kruskal–Wallis with Dunn’s tests for post hoc comparisons (d22) were performed on 2^−ΔΔct^ values.

## 3. Results

### 3.1. Production Performance

All animals remained healthy throughout the study period, with no mortality, removals, or exclusions; all pigs assigned to each dietary group were retained for data collection and included in the statistical analyses. No adverse effects of GOS or XOS supplementation were detected throughout the study, with prebiotic-fed pigs displaying normal feed intake and growth patterns comparable to historical performance at the Harper Adams University Future Farm Pig Unit.

Pig BW at days 0, 7, 22, and 54, as well as ADG and ADFI, were normally distributed based on Shapiro–Wilk tests (*p* > 0.05), whereas BW on d14 and FCR were not (*p* < 0.05). There were no significant differences in BW between diet groups at any timepoint (*p* > 0.05; *t*-test, Kruskal–Wallis tests; Table 2); however, BW on d14 approached significance (*p* = 0.054; Kruskal–Wallis test), with CON pigs (11.61 kg) heavier than GXOS (10.94 kg) and XOS (10.92 kg) pigs. The ADG, ADFI, and FCR did not differ significantly between diet groups across the study period (*p* > 0.05; *t*-test, Kruskal–Wallis tests).

### 3.2. GIT Microbiota Diversity

A total of 22,082,685 high-quality V4 16s rRNA sequence reads were obtained from 432 piglet GIT samples. Subsampling to 18,963 reads per sample achieved a Good’s coverage of 99.2–99.9% (minimum to maximum across all samples). Rarefaction curves generated by resampling without replacement approached asymptotes for all samples, confirming that sequencing depth was sufficient (Figure S1).

**Figure 1:**
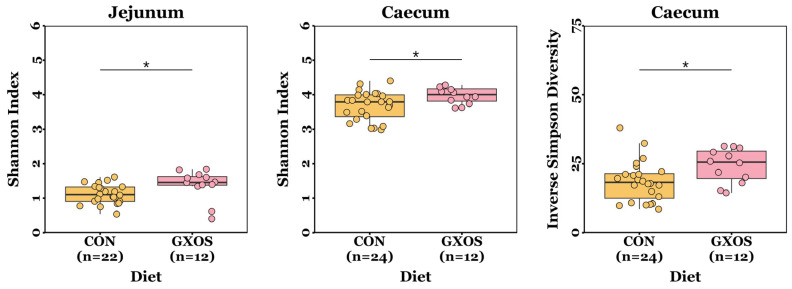
Differences in microbiota α-diversity between CON and GXOS pigs on day 7 post-weaning. *, *p* < 0.05; Wilcoxon rank sum tests. Boxplots are presented as median, Q1 and Q3, while whiskers extend from the smallest value ≥ Q1 − 1.5 × IQR up to the greatest value ≤ Q3 − 1.5 × IQR.

The ACE, Chao richness, Shannon index, and inverse Simpson diversity measurements were normally distributed for most samples (*p* > 0.05; Shapiro–Wilk tests), but not all. Jejunal and caecal Shannon diversity indices were significantly greater (*p* = 0.013 and *p* = 0.035, respectively; Wilcoxon rank sum tests), and caecal inverse Simpson diversity was significantly greater (*p* = 0.022; Wilcoxon rank sum test), in GXOS pigs compared to controls on d7 (Figure 1). There were no significant differences (*p* > 0.05; Wilcoxon rank sum and Kruskal–Wallis tests) in ACE or Chao richness measurements between diet groups throughout the study (Table S1).

**Figure 2:**
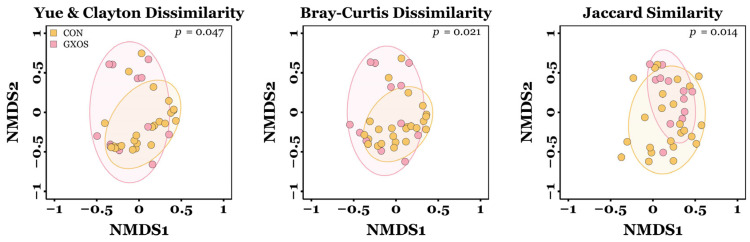
Differences in microbiota β-diversity of ileal samples between CON and GXOS pigs on day 7 post-weaning. CON, *n* = 24; GXOS, *n* = 12. Coloured ellipses represent the 75% confidence interval around the centroid of each treatment group.

Significant differences in the microbial β-diversity of ileal communities were observed between CON and GXOS pigs on d7 for Yu and Clayton (*p* = 0.047), Bray–Curtis (*p* = 0.021), and Jaccard similarity (*p* = 0.014) distances, as determined by AMOVA (Figure 2). No other significant differences in β-diversity were observed during the trial (*p* > 0.05; Figure S2; Table S2).

### 3.3. GIT Microbiota Composition

**Figure 3:**
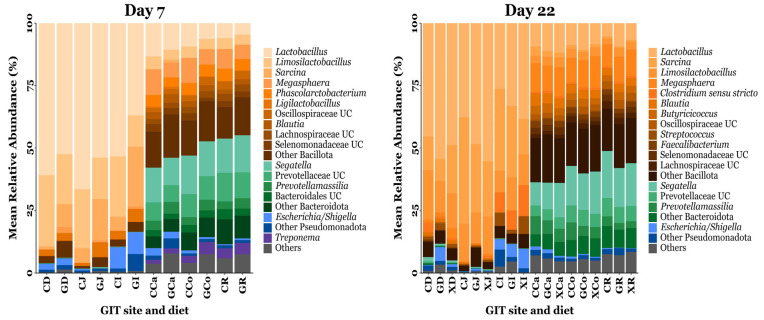
The mean relative abundance of bacterial taxa annotated to OTUs identified from the GIT digesta of piglets fed a control or prebiotic-supplemented diet on day 7 and day 22 post-weaning. The exact mean relative abundance and standard error of the mean for each taxon is provided in Tables S8 and S9. UC = unclassified. CD = CON duodenum; GD = GXOS duodenum; XD = XOS duodenum; CJ = CON jejunum; GJ = GXOS jejunum; XJ = XOS jejunum; CI = CON ileum; GI = GXOS ileum; XI = XOS ileum; CCa = CON caecum; GCa = GXOS caecum; XCa = XOS caecum; CCo = CON colon; GCo = GXOS colon; XCo = XOS colon; CR = CON rectum; GR = GXOS rectum; XR = XOS rectum. Sample sizes for each day, GIT location, and diet group are as follows: day 7—duodenum (CON: *n* = 23, GXOS: *n* = 11), jejunum (CON: *n* = 22, GXOS: *n* = 12), ileum (CON: *n* = 24, GXOS: *n* = 12), caecum (CON: *n* = 24, GXOS: *n* = 12), colon (CON: *n* = 21, GXOS: *n* = 11), rectum (CON: *n* = 20, GXOS: *n* = 10); day 22—duodenum (CON: *n* = 9, GXOS: *n* = 8, XOS: *n* = 8), jejunum (CON: *n* = 9, GXOS: *n* = 10, XOS: *n* = 8), ileum (CON: *n* = 12, GXOS: *n* = 12, XOS: *n* = 11), caecum (CON: *n* = 12, GXOS: *n* = 12, XOS: *n* = 12), colon (CON: *n* = 10, GXOS: *n* = 12, XOS: *n* = 12), rectum (CON: *n* = 10, GXOS: *n* = 10, XOS: *n* = 12).

A total of 7286 OTUs were clustered and taxonomically classified into 25 phyla, 50 classes, 103 orders, 198 families, and 503 genera. On d7, the predominant phyla were Bacillota (72.38%), Bacteroidota (18.68%), Pseudomonadota (4.28%), and Spirochaetota (2.12%), while unclassified bacteria comprised 0.97% of the total sequences. By d22, Bacillota remained dominant (76.11%), followed by Bacteroidota (16.49%), Pseudomonadota (3.48%), and Campylobacterota (1.19%), while unclassified bacteria accounted for 0.37% of the total sequences. The most abundant genera identified on d7 were *Lactobacillus* (31.17%), *Limosilactobacillus* (12.62%), *Sarcina* (5.32%), *Megasphaera* (4.17%), *Phascolarctobacterium* (2.28%), *Ligilactobacillus* 1.94%), *Oscillospiraceae* unclassified (1.35%), *Blautia* (1.29%), *Lachnospiraceae* unclassified (1.24%), and *Selenomonadaceae* unclassified (1.23%). By d22, the predominant genera were *Lactobacillus* (26.06%), *Sarcina* (12.38%), *Limosilactobacillus* (8.46%), *Megasphaera* (5.86%), *Clostridium sensu stricto* (2.28%), *Blautia* (2.13%), *Butyricicoccus* (1.55%), *Oscillospiraceae* unclassified (1.40%), *Streptococcus* (1.30%), and *Faecalibacterium* (1.16%). The relative abundances of the predominant bacterial taxa identified throughout the pig GIT are summarised in Figure 3.

**Figure 4:**
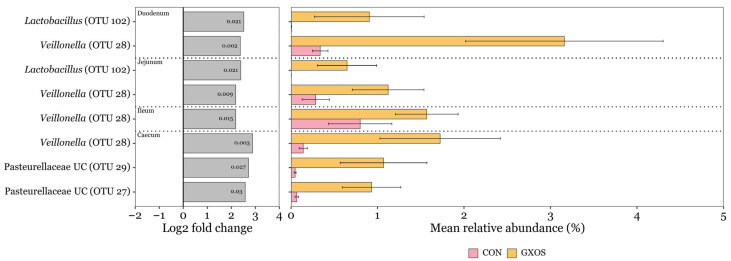
Discriminant OTUs between control and GXOS pigs identified by ANCOM-BC2 on day 7 post-weaning. Only OTUs with ≥ 1% relative abundance in ≥ 1 sample are shown. Log_2_ fold change bars and *p*-values inside log_2_ fold change bars were obtained from ANCOM-BC2 analysis. Mean relative abundance bars are the average of within sample relative abundances per diet and GIT section; error bars represent standard error of the mean. Sample sizes for each GIT location and diet group are as follows: duodenum (CON: *n* = 23, GXOS: *n* = 11), jejunum (CON: *n* = 22, GXOS: *n* = 12), ileum (CON: *n* = 24, GXOS: *n* = 12), caecum (CON: *n* = 24, GXOS: *n* = 12).

ANCOM-BC2 was used to identify significant differences in the differential abundance of OTUs assigned to bacterial taxa at the genus level, applying a minimum prevalence threshold of 10% and a minimum sequencing depth of 50. On d7, a total of eight OTUs were significantly more abundant (*p* < 0.05) in GXOS pigs compared to controls, with two enriched in the duodenum, two in the jejunum, one in the ileum, and three in the caecum (Figure 4). *Veillonella* (OTU 28) was enriched across all four of these regions, while *Lactobacillus* (OTU 102) was significantly enriched in duodenal and jejunal communities. No OTUs were depleted by GXOS on d7 across the GIT. Additionally, no OTUs were significantly differentially abundant in GXOS or XOS pigs compared to controls on d22, according to ANCOM-BC2 analysis.

**Figure 5:**
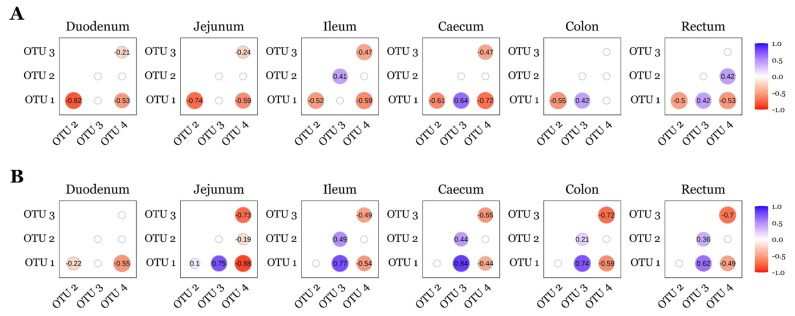
Correlations between the top four OTUs observed across the pig GIT for day 7 (**A**) and day 22 (**B**) post-weaning. OTUs were identified as follows: *Lactobacillus* (OTU 1); *Lactobacillus* (OTU 2); *Limosilactobacillus* (OTU 3); *Sarcina* (OTU 4). A positive number denotes positive correlation (blue); a negative number denotes a negative correlation (red); insignificant Spearman’s rank analyses (*p* > 0.05) are presented as blank nodes. Sample sizes for each day and GIT location are as follows: d7—duodenum *n* = 34, jejunum *n* = 34, ileum *n* = 36, caecum *n* = 36, colon *n* = 32, rectum *n* = 30; d22—duodenum *n* = 25, jejunum *n* = 27, ileum *n* = 35, caecum *n* = 36, colon *n* = 34, rectum *n* = 32.

*Lactobacillus* (OTU 1), *Lactobacillus* (OTU 2), *Limosilactobacillus* (OTU 3), and *Sarcina* (OTU 4) were not differentially abundant (*p* > 0.05; ANCOM-BC2) between diet groups for any timepoint or GIT section. Therefore, relative abundances were pooled across diets and correlation analyses were stratified by timepoint and intestinal site. Spearman’s rank correlation analysis revealed consistent interactions among these OTUs across GIT sites and timepoints (Figure 5). On d7, *Lactobacillus* (OTU 1) was negatively correlated with *Lactobacillus* (OTU 2) and *Sarcina* (OTU 4) in the duodenum, jejunum, ileum, caecum and rectum. *Limosilactobacillus* (OTU 3) was negatively correlated with *Sarcina* (OTU 4) in the duodenum, jejunum, ileum, and caecum. In the ileum, *Lactobacillus* (OTU 2) showed a positive correlation with Limosilactobacillus (OTU 3), while in the caecum, colon, and rec- tum, *Lactobacillus* (OTU 1) was positively correlated with *Limosilactobacillus* (OTU 3). On d22, *Lactobacillus* (OTU 1) remained negatively correlated with *Sarcina* (OTU 4) across all GIT sites. Positive correlations between *Lactobacillus* (OTU 1) and *Limosilactobacillus* (OTU 3) were observed in the jejunum, ileum, caecum, colon, and rectum. Additionally, *Lactobacillus* (OTU 2) and *Limosilactobacillus* (OTU 3) were positively correlated in the ileum, caecum, colon, and rectum, while *Limosilactobacillus* (OTU 3) was negatively correlated with *Sarcina* (OTU 4) in the jejunum, ileum, caecum, colon, and rectum.

### 3.4. Histology and Gut Architecture

**Figure 6:**
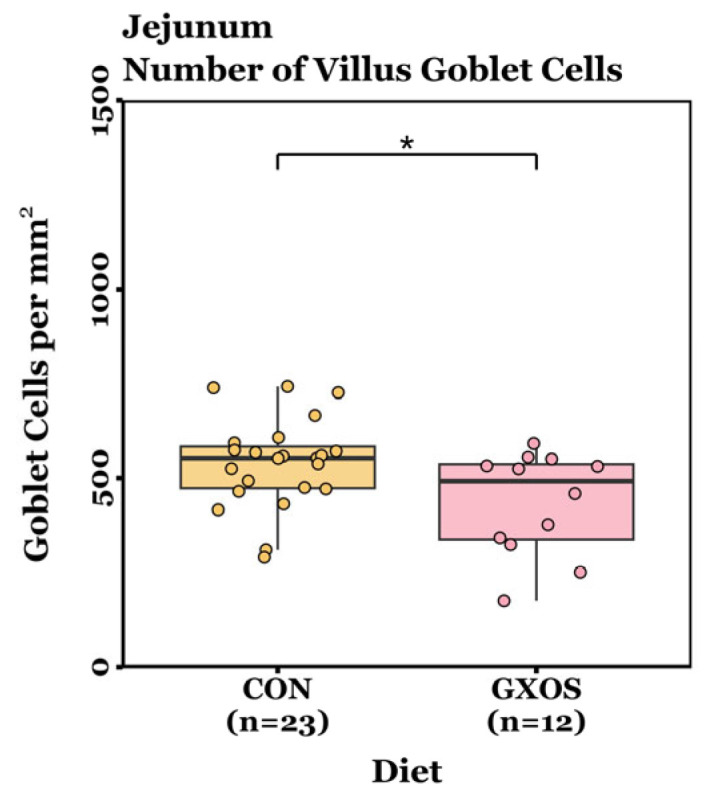
Differences in jejunal GC density on day 7 post-weaning between CON and GXOS groups. Wilcoxon rank sum test; *, *p* < 0.05. Boxplots are presented as median, Q1 and Q3, while whiskers extend from the smallest value ≥ Q1 − 1.5 × IQR up to the greatest value ≤ Q3 − 1.5 × IQR.

Villus height, crypt depth, VCR, and GC density measurements were not normally distributed (*p* < 0.05, Shapiro–Wilk tests) and were therefore analysed using non-parametric Wilcoxon rank-sum tests (day 7) or Kruskal–Wallis tests followed by Dunn’s post hoc test for pairwise comparisons (day 22). On d7, no significant differences in villus height, crypt depth, VCR, or crypt GC density were detected between CON and GXOS-fed pigs across the jejunum and ileum (*p* > 0.05; Wilcoxon rank sum tests; Figure S3 and Table S3). However, jejunal villus GC density was significantly reduced in GXOS pigs compared to controls (*p* = 0.028; Wilcoxon rank sum test; Figure 6). On d22, no significant differences were detected in villus height, crypt depth, VCR, villus GC density, or crypt GC density among the CON, GXOS, and XOS groups across the jejunum and ileum (*p* > 0.05; Kruskal–Wallis tests; Figure S3 and Table S3).

### 3.5. Immunomodulation

**Figure 7:**
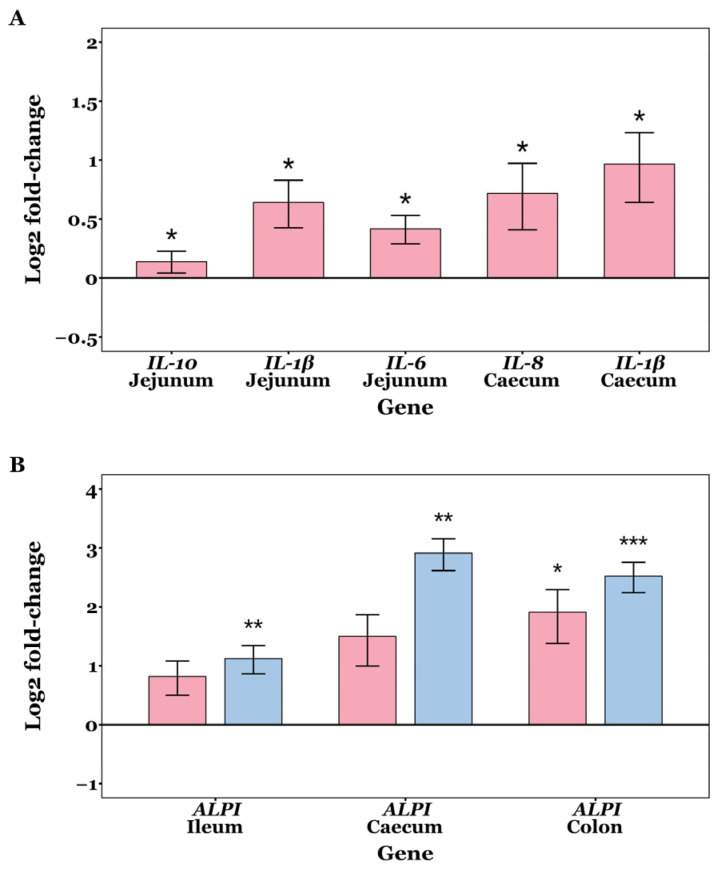
Changes in expression of *IL-1β*, *IL-6*, *IL-8*, and *ALPI* on day 7 (**A**) and day 22 (**B**) post-weaning in piglet GIT tissues across CON, GXOS and XOS diets. Wilcoxon rank sum tests (day 7); Kruskal–Wallis and Dunn’s tests (day 22). Gene expression was measured by qRT-PCR. Relative fold changes were calculated using the 2^−∆∆ct^ method, followed by log_2_ transformation; values for the CON group are zero by definition and omitted from the plots, such that deviations from the *x*-axis represent up- or downregulation. Expression of target genes was normalised to the average of *RPL4* and *GAPDH* within each sample. Bars indicate log_2_(fold change) for GXOS (pink) and XOS (blue) diet groups. Asterisks directly above bars denote significant differences relative to the CON group. For each sample and gene, ct values >35 were excluded from analysis, and outliers were removed using the 1.5 × IQR method. Sample sizes for each gene and diet group presented are as follows: day 7—jejunum: IL-10 (CON: *n* = 21, GXOS: *n* = 11), IL-1β (CON: *n* = 21, GXOS: *n* = 12), IL-6 (CON: *n* = 21, GXOS: *n* = 10), caecum: IL-8 (CON: *n* = 21, GXOS: *n* = 10), IL-1β (CON: *n* = 22, GXOS: *n* = 12); day 22—ALPI: ileum (CON: *n* = 10, GXOS: *n* = 12, XOS: *n* = 12), caecum (CON: *n* = 10, GXOS: *n* = 11, XOS: *n* = 11), colon (CON: *n* = 10, GXOS: *n* = 11, XOS: *n* = 12). *, *p* < 0.05; **, *p* < 0.01; ***, *p* < 0.001.

Fold change values were normally distributed in most samples (Shapiro–Wilk tests *p* > 0.05), but not all. No significant differences were observed in the mRNA expression levels of *OCLN*, *CLDN2*, *CLDN3*, *ZO-1*, and *ZO-2* among the CON, GXOS and XOS groups across GIT sites throughout the study (*p* > 0.05; Wilcoxon rank sum and Kruskal–Wallis tests). On d7, jejunal expression of *IL-1β* (*p* = 0.014), *IL-6* (*p* = 0.025) and *IL-10* (*p* = 0.032) was significantly upregulated in GXOS pigs compared to controls (Wilcoxon rank sum tests). In the caecum, *IL-1β* (*p* = 0.044) and *IL-8* (*p* = 0.048) expression was also significantly increased in the GXOS group (Wilcoxon rank sum tests). On d22, *ALPI* expression was significantly upregulated in ileal (*p* = 0.008) and caecal (*p* = 0.004) tissues of XOS pigs, while *ALPI* expression was significantly upregulated in colonic tissues of both GXOS (*p* = 0.043) and XOS (*p* = 0.001) groups, relative to controls (Kruskal–Wallis tests). The effects of GXOS and XOS supplementation on target gene expression are shown in Figure 7.

Correlation analysis revealed that *Sarcina* (OTU 4) negatively correlated with *OCLN* expression in the jejunum on d7 (r_s_ = -0.422, *p* = 0.047; Spearman’s rank correlation).

## 4. Discussion

This study investigated whether sequential supplementation with GOS immediately post-weaning, followed by prolonged XOS feeding, could enhance intestinal health and performance in healthy weaning piglets. The approach was designed to reflect the natural dietary transition from milk oligosaccharides to xylan-rich cereal carbohydrates, allowing early GOS exposure to stabilise the weaning microbiota and support later XOS fermentation. Although sow’s milk and commercial GOS differ structurally [34,52], both act as fermentable substrates for beneficial taxa such as *Lactobacillus* and *Bifidobacterium* [35,36,37,38,39], which may facilitate microbial adaptation across the dietary transition.

In the present study, GXOS and XOS supplementation had no effect on performance metrics: no significant differences were observed in BW at days 7, 14, 22, or 54, nor in ADG or FCR over days 1–54 between diet groups (Table 2). Additionally, ADFI did not differ significantly between diet groups, indicating that a lack of improved BW and ADG was not due to differences in gross energy intake. In contrast to our seven-day GOS pre-conditioning, Tian et al. (2018) [37] reported enhanced ADG when GOS was administered to pre-weaning piglets during their first week of life, while Xing et al. (2020) [38] observed improvements in both ADG and FCR following 28 days of post-weaning GOS supplementation, indicating that early-life or prolonged post-weaning exposure to GOS is required to realise performance benefits. However, Lee et al. (2023) [68] found that unthrifty piglets supplemented with GOS in milk replacer showed no significant gains in body weight or ADG, suggesting that GOS efficacy in improving growth performance may depend on host health status or require longer supplementation in unthrifty or compromised animals. By d54, performance metrics did not differ among the CON, GXOS, and XOS groups. Contrastingly, healthy piglets weaned at 28 or 30 days and supplemented with XOS for 28 days are reported to exhibit improved body weight, ADG, and gain-to-feed ratio [43,45], though these studies were conducted in different crossbreeds than those used here, which may contribute to differences in performance. In the present study, the absence of enhanced performance of prebiotic groups, as well as their near-significant reduction in BW on day 14, may reflect mismatches between substrate availability and microbial adaptation: in the sequential GXOS diet group, early GOS exposure may have primed communities that then required additional time to adjust to XOS, delaying microbial shifts and subsequent XOS-mediated performance benefits; conversely, in the XOS group, introducing XOS after seven days of the basal diet may have disrupted the stabilising microbial community, requiring a reorganisation of established cross-feeding networks and imposing short-term metabolic costs that limited growth efficiency. Moreover, the subsequent 47-day period of XOS feeding after this transition may have been insufficient to produce measurable performance gains by day 54 relative to control pigs that maintained an uninterrupted microbial trajectory, particularly as the delay in XOS introduction likely missed the critical post-weaning window during which the microbiota is most susceptible to dietary modulation.

Significant increases in the microbiota α-diversity were observed on d7 in the GOS-fed group compared to controls (Figure 1): both jejunal and caecal Shannon indices, as well as caecal inverse Simpson diversity, were greater, indicating that GOS supplementation enhanced microbial richness and evenness. Previous studies have reported that GOS supplementation in healthy weaning piglets increases colonic mucosal ACE and Chao1 indices [39], whereas GOS had no impact on ACE, Chao1, Shannon, or Simpson metrics in pigs challenged with *Escherichia coli* lipopolysaccharide (LPS) [40]. By d22, α-diversity no longer differed among GXOS, XOS, and CON groups, indicating that the GOS-induced elevation of richness and evenness was not maintained with XOS supplementation. This likely reflects the inability of the GOS-adapted microbial community—comprising lactose- and galactose-degrading bacteria—to rapidly adapt to XOS metabolism within the short 15-day supplementation period, thereby preventing continued proliferation of established taxa once GOS was withdrawn. A longer XOS feeding period may have allowed the establishment of XOS-degrading microbiota and greater α–diversity, as reported in previous studies which report increased ACE, Chao1, and Shannon indices in healthy piglets receiving XOS at weaning for 28 days [42,84]. Alternatively, the convergence in α-diversity across groups may reflect natural post-weaning microbiota maturation rather than a direct dietary effect. Nevertheless, these findings underscore the importance of prebiotic timing and duration at weaning to support sustained microbial diversity.

AMOVA of ileal communities on d7 revealed significant β-diversity differences between CON and GXOS pigs for the Yue and Clayton, Bray–Curtis, and Jaccard indices (Figure 2). By d22, β-diversity did not differ among CON, GXOS, and XOS groups, indicating that GOS-induced community shifts were not sustained during XOS supplementation. Previous findings demonstrate that individual administration of GOS [37,39] or XOS [8,42,85] modulates GIT microbial composition in healthy pre- and post-weaning piglets. In the present study, replacing GOS with XOS appeared to disrupt the GOS-adapted microbial community, and the subsequent 15-day XOS phase did not allow sufficient time for XOS-utilising populations to establish a stable community structure.

The variations observed in α- and β-diversity across intestinal segments likely reflect physiological and ecological gradients along the GIT and the interaction between prebiotic substrate availability and the inherent microbial richness of each site. The duodenum, being acidic, oxygenated, and characterised by rapid transit [86,87], supports limited microbial colonisation, which likely explains the absence of diversity changes. The jejunum provides a more favourable environment for fermentation, with slower transit and higher bacterial abundance [86,87], where GOS likely acted as an additional substrate supporting the proliferation of commensals that were not strongly stimulated by the basal diet alone, leading to greater α-diversity. In the ileum, which contains a more complex microbial community than the jejunum [87], the GOS-induced increases in richness may be masked by the higher baseline diversity; instead, compositional restructuring (β-diversity shifts) was observed, modulating which microbes dominate rather than increasing overall richness. In the caecum, exposure to newly available complex substrates and longer retention times likely allowed GOS to further stimulate fermentative cross-feeding networks and enhance α-diversity, though β-diversity remained unchanged—probably because the caecal microbiota is already dominated by a stable microbial community, meaning GOS increased richness among existing taxa rather than altering their dominance. No diversity changes were detected in the colon or rectum, which may reflect substrate depletion prior to reaching these distal sites.

A total of 25 phyla were detected across all pig GIT microbial communities throughout the study, comprising 7286 OTUs. Of these, Bacillota (formerly Firmicutes) dominated the small intestine (Figure 3), while the large intestine was co-dominated by Bacillota and Bacteroidota (formerly Bacteroidetes), in keeping with previous results in similar-aged weaning pigs [48]. Within the small intestine, Bacillota—primarily the genera *Lactobacillus* and *Limosilactobacillus* in this study—thrive in the acidic gut lumen and possess phosphotransferase systems for efficient uptake of simple sugars [88,89]. By contrast, complex plant polysaccharides resist small-intestinal digestion and reach the large intestine, where Bacteroidota ferment these substrates via diverse carbohydrate-degrading enzymes [25]. Differentially abundant genera were identified between diet groups using ANCOM-BC2 (Figure 4). At d7, GXOS pigs exhibited increased *Lactobacillus* (OTU 102) in the duodenum and jejunum, and increased *Veillonella* (OTU 28) across the duodenum, jejunum, ileum, and caecum, relative to controls. By d22, neither the GXOS or XOS group exhibited any differentially abundant OTUs, indicating that the taxa enriched by GOS at d7 were not sustained and that XOS supplementation alone did not significantly enrich or deplete any taxa, consistent with the α- and β-diversity observations in this study (Figure 1 and Figure 2). *Lactobacillus* spp. ferment dietary carbohydrates to produce acetic, lactic, propionic and butyric acids, thereby lowering the GIT lumen pH and inhibiting pathogen colonisation [27,90], while *Veillonella* spp. cross-feed on lactate produced by primary fermenters and convert it mainly to propionate, with acetate as a co-product, via the succinate pathway, thereby contributing to the luminal SCFA pool [91,92]. The concurrent enrichment of these taxa at d7 therefore suggests enhanced metabolic cross-feeding and a potential increase in luminal SCFA production—particularly propionate—during GOS supplementation, though SCFA concentrations were not directly measured in this study. These SCFAs serve as energy sources for enterocytes, supporting tight junction integrity, thereby strengthening the gut barrier [93]. Previous work consistently shows that GOS supplementation enriches *Lactobacillus* abundance across both in vitro and in vivo piglet systems—from large intestine fermentation models inoculated with porcine microbiota [94] to healthy weanling pigs [36,38], unthrifty neonates [68], and *S*. *typhimurium*-challenged piglets [41]. Similarly, XOS has been reported to promote *Lactobacillus* abundance in the ileum [42,53], caecum [42,95], and faeces [44,45] of healthy weanlings, in the colon of healthy fattening pigs [96], and in the duodenum and jejunum of diarrhoeic post-weaning piglets [48]. Notably, these studies administered XOS for 22–28 days in weanling trials or up to 100 days in fattening trials—far longer than the 15-day XOS phase used here—which may explain why no significant shifts in *Lactobacillus* or other taxa were observed across diet groups in this study. Moreover, although beneficial taxa were enriched by GOS, their persistence—and that of associated cross-feeding networks—depends on continued access to their preferred carbohydrate sources. Consequently, when GOS is withdrawn and XOS introduced, the resource availability changes, and GOS-adapted taxa decline more rapidly than XOS-utilising populations can become established.

*Lactobacillus* (OTU 1) and *Limosilactobacillus* (OTU 3) were consistently negatively correlated with *Sarcina* (OTU 4) across all GIT sites at both d7 and d22 (Figure 5), whereas *Lactobacillus* (OTU 2) displayed more variable relationships—positively correlating with *Limosilactobacillus* (OTU 3) across several sites but showing weaker or site-specific associations with *Sarcina* (OTU 4). Two porcine gut enterotypes have been proposed, with one dominated by *Lactobacillus* and another by *Sarcina* co-dominating with additional genera [97,98]. In those studies, the *Sarcina*-dominated enterotype exhibited lower α-diversity, but BW, ADG, ADFI, and FCR at slaughter age did not differ between enterotypes, suggesting that enterotype alone is not predictive of growth performance [97,98]. Consistent with reports that higher *Sarcina* abundance is associated with reduced Lactobacillaceae and Bifidobacteriaceae [99,100], suppression of *Sarcina* by lactic acid bacteria may be beneficial in the pig gut; *Sarcina* has been linked to acute gastric dilatation in dogs and horses, and to delayed gastric emptying, emphysematous gastritis, and gastric perforation in humans [101,102,103]. In our study, however, evidence for an inflammatory association was limited: we detected a single negative correlation between *Sarcina* (OTU 4) and *OCLN* mRNA in the jejunum on d7 but did not observe any further or consistent associations between *Sarcina* (OTU 4) and transcripts encoding other tight junction proteins, cytokines, or chemokines.

Dietary intervention had a transient impact on gut morphological responses (Figure 6). Jejunal villus GC density was significantly reduced in GXOS pigs after 7 days, but this effect was not sustained by d22 following XOS supplementation, a trend consistent with the microbiota findings in this study. Weaning has been shown to activate innate and inflammatory signalling pathways, such as NOD-like receptors, Toll-like receptors (TLRs), and JAK-STAT, compared with unweaned counterparts [13]. Consequently, post-weaning piglets exhibit upregulated expression of *IL-1β*, *IL-6*, *IL-8*, and *TNF-α* during the first week of weaning [7,8]. These cytokines can stimulate regulated mucin exocytosis from goblet cells, as demonstrated in duodenal explant models where IL-1 and IL-1β triggered mucus release [104,105], and in HT29-MTX cells where TNF- α, IL-6, and bacterial LPS upregulated mucin gene expression and secretion [106,107]. In the present study, the elevated cytokine expression observed in GOS-fed pigs may have accelerated mucin secretion, leading to depletion of intracellular mucin granules and, consequently, fewer PAS-positive goblet cells. Because PAS staining primarily detects stored mucins, the apparent reduction in GC density may reflect empty or depleted cells resulting from increased secretory activity, characteristic of mucosal priming, rather than an actual loss of goblet cell populations. Dietary prebiotic supplementation had no effect on villus length during this study. Given that migration of intestinal epithelial cells from the crypt base to the villus tip takes approximately 3–5 days, the 7-day GOS feeding period followed by only 15 days of XOS likely did not provide sufficient time for the microbiota to adapt to the substrate switch and for downstream morphological changes to develop [108,109]. This highlights the importance of aligning prebiotic intervention length with epithelial turnover kinetics, as structural and functional adaptations within the mucosa may only become apparent after several renewal cycles. Future studies should therefore extend the XOS feeding phase to determine the optimal duration for sustained mucosal and microbial adaptation. Previous studies report that GOS or XOS supplementation is associated with increased villus height, VCR, and GC density in unthrifty pre-weaning [68], healthy post-weaning [36,42,43], and diarrhoeic post-weaning [48] piglets. By way of comparison, these trials supplemented a single prebiotic continuously for 22–28 days, without substrate switching, providing sustained trophic stimulation across multiple epithelial turnover cycles.

On d7, GOS pre-conditioning increased jejunal *IL-1β*, *IL-6*, and *IL-10*, and caecal *IL-1β* and *IL-8* relative to controls (Figure 7). Weaning is known to activate innate immune and inflammatory signalling pathways, during the first week postweaning [13]. Accordingly, an inflammatory increase would be expected across all diets at day 7; however, the greater α-diversity observed in the jejunum and caecum of GOS-fed pigs (Figure 1) may have increased the variety of microbe-/pathogen-associated molecular patterns (MAMPs/PAMPs), thereby activating pathogen recognition receptor (PRR) signalling (e.g., TLR/NF-κB) and transiently elevating *IL-1β*, *IL-6*, and *IL-8*. The enrichment of *Lactobacillus* and *Veillonella* at d7, and their potential lactate-propionate cross-feeding, may have increased luminal SCFA availability; SCFAs signal via FFAR2/FFAR3 (GPR43/GPR41) and HDAC inhibition to promote anti-inflammatory IL-10 induction and support epithelial energy supply and tight junction maintenance, which may explain the concurrent rise in *IL-10* at d7 as the mucosa sought to restore homeostasis [110,111]. In this respect, the increased inflammatory activity associated with the 7-day GOS pre-conditioning may indicate transient mucosal priming rather than sustained inflammation and likely represents a trade-off accompanying community diversification, since greater α-diversity is associated with improved long-term performance [48,112], enrichment of health-associated taxa [113], enhanced barrier integrity [114], and greater colonisation resistance [48]. By d22, after 15 days of XOS, the weaning-associated inflammatory signalling had disappeared, while only *ALPI* was elevated in the ileum and caecum for the XOS group, and in the colon for both GXOS and XOS groups, suggesting a potential shift toward a more barrier-protective mucosal state. Dephosphorylation of the bacterial LPS lipid A moiety by ALPI dampens TLR4 activation and downstream NF-κB signalling, attenuating inflammatory cascades that would otherwise compromise tight junctions, increase paracellular permeability, and promote antigen translocation [115,116]. Collectively, the d7 cytokine profile—elevated *IL-1β*, *IL-6*, *IL-8*, and *IL-10*—is consistent with transient mucosal priming rather than sustained inflammation. This is supported by (i) resolution of pro-inflammatory cytokine differences by d22; (ii) concurrent induction of *IL-10* (rather than unilateral pro-inflammatory up-regulation), and (iii) subsequent *ALPI* up-regulation, which is indicative of dampened TLR4–NF-κB signalling. Together with the absence of histological injury and normalisation of GC density by d22, these findings indicate short-lived innate activation followed by a barrier-protective homeostatic state, rather than progression to chronic inflammation. Previous studies have shown that *ZO-1* and *ZO-2* expression is decreased in *ALPI*-knockout mice, and that *ALPI* expression is reportedly decreased in early-weaned piglets compared to suckling counterparts [117,118]. Consistent with our findings, *ALPI* expression reportedly increases in XOS-supplemented post-weaning diarrhoeic pigs after 22 days [48], further demonstrating XOS’s role in alleviating LPS-induced inflammation and maintaining intestinal homeostasis. Although direct evidence for XOS effects on *ALPI* is limited, other non-digestible oligosaccharides (GOS, fructo-oligosaccharides, raffinose, lactulose) have been shown to increase *ALPI* expression in rats [119], supporting a general role for fermentable oligosaccharides in maintaining intestinal homeostasis.

Recent studies reinforce the potential of prebiotic strategies to improve gut health in weaned piglets: supplementation with either GOS [37,38] or XOS [42,53,54] is reported to enhance growth performance through GIT microbiota modulation and to improve gut health through beneficial changes in intestinal architecture and epithelial barrier function. Although direct trials combining GOS and XOS within the same post-weaning ration remain absent, the complementary modes of action observed across these studies align with our sequential approach and indicate that sequential GOS–XOS—or concurrent formulations—could stabilise the microbiota, promote barrier integrity, and reduce reliance on antibiotics by improving mucosal resilience. Future studies should evaluate transition strategies that minimise perturbation from prebiotic switching post-weaning, for example, a brief GOS–XOS overlap/taper rather than an abrupt supplement change. Synbiotic designs should also be tested at both phases: GOS + probiotic during the early priming window (to establish *Lactobacillus*/*Bifidobacterium* and seed lactate producers), followed by XOS + probiotic during the maintenance phase to sustain established cross-feeding networks. For instance, pairing XOS with a targeted *Lactobacillus* strain (e.g., *Lactobacillus plantarum* B90 [120]) in this study could have helped maintain the GOS-established communities and translate transient mucosal priming into performance gains. Collectively, these approaches will help optimise non-antibiotic feeding strategies by defining conditions under which prebiotic (and synbiotic) programs convert early microbial priming into durable barrier support and measurable productivity gains in commercial pig production systems.

## 5. Conclusions

We have demonstrated that dietary supplementation of 1% GOS to 28-day old healthy weaning pigs for 7 days transiently modulates the gut microbiota by increasing jejunal and caecal α-diversity, shifting ileal β-diversity, and enriching SCFA-producing *Lactobacillus* and *Veillonella*. However, these changes were not maintained after switching to 0.017% XOS supplementation; by d22 there were no differences in α- or β-diversity, nor were any taxa differentially abundant in GXOS or XOS pigs. Performance (BW, ADG, ADFI, FCR) did not differ between diets throughout the study. A transient reduction in jejunal GC density was observed at d7, but no sustained morphological effects were observed by d22. On d7, GOS was associated with elevated *IL-1β*, *IL-6*, *IL-8*, and *IL-10* expression in the jejunum and caecum, consistent with short-term, diversity-linked mucosal priming. By d22 cytokine/chemokine differences had resolved, and *ALPI*, a brush border enzyme that dephosphorylates bacterial LPS to reduce TLR4 activation, NF-κB signalling, and gut permeability, was upregulated in the ileum, caecum, and colon by XOS, indicating a greater barrier-protective state. Collectively, these findings suggest that prebiotic timing and duration are critical: a brief GOS phase can diversify communities, but sequential prebiotic switching and delayed XOS onset likely misses the immediate post-weaning window required to produce microbial and mucosal changes that impose gains in performance.

## Figures and Tables

**Table 1:** Formulation and nutrient composition of the basal diet for day 1–day 7, day 8–day 22, and day 23–day 54 post-weaning.

Item	Day 1–Day 7	Day 8–Day 22	Day 23–Day 54
Ingredient (inclusion %)			
Micronised barley	10.00	15.00	0.00
Barley	0.00	0.00	15.00
Wheat (raw whole meal)	20.44	37.91	47.87
Micronised wheat (meal)	10.00	5.00	0.00
Wheatfeed	0.00	0.00	1.03
Micronised oats	10.00	0.00	0.00
Fishmeal	7.25	5.77	0.00
Hypro soya bean meal	19.00	24.00	27.10
Premix 1 ^1^	0.50	0.50	0.00
Dried skim milk	4.00	0.00	0.00
Whey powder	11.41	7.25	3.62
L-lysine HCl	0.312	0.236	0.483
L-methionine	0.19	0.124	0.189
L-threonine	0.19	0.121	0.215
L-tryptophan	0.029	0.00	0.009
L-valine	0.066	0.00	0.10
Vitamin E	0.02	0.01	0.03
Sucram	0.01	0.01	0.01
Dicalcium phosphate	0.84	0.97	1.95
Soya oil	5.74	2.94	1.68
Pure dried vacuum salt	0.00	0.16	0.41
Premix 2 ^2^	0.00	0.00	0.25
Copper sulphate	0.00	0.00	0.03
Iron (II) chelate of glycine	0.00	0.00	0.03
Nutritional composition (calculated %)			
Dry matter	90.57	89.19	88.48
Moisture	9.43	10.81	11.52
Oil A ^3^	8.00	4.67	3.03
Oil B ^4^	8.80	5.48	3.88
Crude protein (N × 6.25)	22.19	21.87	19.97
Fibre	1.98	2.43	2.90
Neutral detergent fibre	6.41	7.91	9.13
Starch	31.25	35.90	38.93
Crude ash	5.48	5.20	5.20
Salt	0.75	0.70	0.75
Calcium	0.87	0.78	0.72
Phosphorus	0.69	0.65	0.71
Digestible phosphorus	0.44	0.39	0.41
Sodium	0.20	0.20	0.20
Zinc (mg/kg)	136.16	135.99	127.97
Copper (mg/kg)	144.97	145.77	96.37
Lysine	1.53	1.39	1.36
Methionine	0.59	0.50	0.47
Net energy (MJ/kg)	10.66	9.73	9.24
Digestible energy (MJ/kg)	15.83	14.83	14.26

^1^ Premix provides per kg of feed: 13,750 IU Vitamin A; 2100 IU Vitamin D3; 150 mg Vitamin E; 6.0 mg Vitamin K3—Menadione; 1.6 mg Vitamin B1; 6.0 mg Riboflavin; 2.3 mg Vitamin B6; 0.028 mg Vitamin B12; 25.0 mg Niacin; 13.3 mg Pantothenic acid; 1.0 mg Folic acid; 0.150 mg Biotin; 1.0 mg Iodine (calcium iodate, anhydrous); 0.25 mg Selenium (Sodium selenite); 150 mg Iron (Iron (II) sulphate monohydrate); 140 mg Copper (Copper (II) sulphate pentahydrate); 110 mg Zinc (Zinc sulphate monohydrate); 40 mg Manganese (Manganous sulphate monohydrate). ^2^ Premix provides per kg of feed: 10,500 IU Vitamin A; 2250 IU Vitamin D3; 50.0 mg Vitamin E; 4.0 mg Vitamin K; 0.874 mg Vitamin K3—Menadione; 1.5 mg Vitamin B1; 4.0 mg Riboflavin; 3.5 mg Vitamin B6; 0.015 mg Vitamin B12; 20.0 mg Niacin; 12.0 mg Calcium pantothenate; 11.035 mg Pantothenic acid; 2.0 mg Folic acid; 0.2 mg Biotin; 1.0 mg Iodine (calcium iodate, anhydrous); 0.25 mg Selenium (Sodium selenite); 80.0 mg Iron (Iron (II) sulphate monohydrate); 15.0 mg Copper (Copper (II) sulphate pentahydrate); 100 mg Zinc (Zinc sulphate monohydrate); 50.0 mg Manganese (Manganous sulphate monohydrate); 10.0 mg Citric acid. Iron (II) chelate of glycine = B-traxim 2c Fe-220, M60-5000. ^3^ Oil A denotes crude oils and fats determined by direct solvent extraction. ^4^ Oil B denotes total crude oils and fats determined after hydrochloric-acid hydrolysis followed by solvent extraction.

**Table 2:** Body weights at wean, d7, d14, d22, and d54, ADG, ADFI, and FCR of control and prebiotic-supplemented post-weaning piglets.

		Diet			Pooled SEM	*p*-Value
Weight, kg	CON		GXOS			
Wean	8.32		8.32		0.01	0.954 ^1^
Day 7	9.54		9.32		0.07	0.117 ^1^
	CON	GXOS		XOS		
Day 14	11.61	10.94		10.92	0.13	0.054 ^2^
Day 22	14.88	14.12		13.95	0.21	0.158 ^3^
Day 54	40.64	38.74		38.03	0.58	0.171 ^3^
ADG, g/d	555.8	525.1		513.9	9.79	0.197 ^3^
ADFI, kg	0.86	0.78		0.79	0.02	0.123 ^3^
FCR	1.55	1.49		1.54	0.02	0.121 ^2^

^1^ *t*-test; ^2^ Kruskal–Wallis test; ^3^ ANOVA. Values are means of the average BW/ADG/ADFI/FCR across the replicate pens for each diet. *n* = 12 pens per diet group.

## Data Availability

The data presented in this study are openly available at https://github.com/J-S-Stanley/GOS-XOS-2025 (accessed on 19 September 2025), with sequence data deposited in the NCBI database within the BioProject PRJNA1308109, with SRA records available at https://www.ncbi.nlm.nih.gov/sra/PRJNA1308109 (accessed on 20 August 2025).

## Supplementary Materials

The following supporting information can be downloaded at: https://www.mdpi.com/article/10.3390/ani15213210/s1. Figure S1: Rarefaction curves showing sequencing effort for all microbiota communities. Figure S2: Supplementary NMDS plots showing β-diversity of GIT microbial communities for control and prebiotic-fed pigs at day 7 and day 22 post-weaning. Figure S3: Complementary boxplots showing histomorphological measurements of control and prebiotic-supplemented pigs at day 7 and day 22 post-weaning. Table S1: Alpha diversity of GIT microbial communities for control and prebiotic-fed pigs at day 7 and day 22 post-weaning. Table S2: Beta diversity showing significant differences in AMOVA between control and prebiotic-fed pigs at day 7 and day 22 post-weaning. Table S3: Histomorphological measurements of control and prebiotic-supplemented pigs at day 7 and day 22 post-weaning. Table S4: Feed formulations of CON, GXOS, and XOS diets used throughout the study. Table S5: Nutritional composition of creep feed offered to all piglets from 15 to 28 days of age. Table S6: Nutritional composition of supplementary milk offered to all piglets from birth to 28 days of age. Table S7: Assay IDs for target and reference genes included in the Qiagen Custom RT^2^ Profiler PCR Array used for quantitative gene expression analysis. Table S8: The mean relative abundance of bacterial taxa from the GIT digesta of piglets fed a control or prebiotic-supplemented diet on day 7 post-weaning. Table S9: The mean relative abundance of bacterial taxa from the GIT digesta of piglets fed a control or prebiotic-supplemented diet on day 22 post-weaning.

## Abbreviations

The following abbreviations are used in this manuscript:

ADFI	Average daily feed intake
ADG	Average daily gain
AGP	Antibiotic growth promoter
ALPI	Intestinal alkaline phosphatase
AMOVA	Analysis of molecular variance
ANOVA	Analysis of variance
BW	Body weight
FCR	Feed conversion ratio
GC	Goblet cell
GIT	Gastrointestinal tract
GOS	Galacto-oligosaccharides
OTU	Operational taxonomic unit
PWD	Post-weaning diarrhoea
PWGC	Post-weaning growth check
SCFA	Short-chain fatty acid
VCR	Villus-to-crypt ratio
XOS	Xylo-oligosaccharides
