# Supplementary material for: Sequential Galacto- and Xylo-Oligosaccharide Feeding Transiently Modulates Gut Microbiota and Upregulates Intestinal Alkaline Phosphatase in Weaning Piglets
Source: Animals (Basel). 2025 Nov 4;15(21):3210. doi: 10.3390/ani15213210 (PMC12609449; doi:10.3390/ani15213210)
Supplement: Supplementary file 1 [file animals-15-03210-s001.zip › animals-3916601-supplementary.pdf]

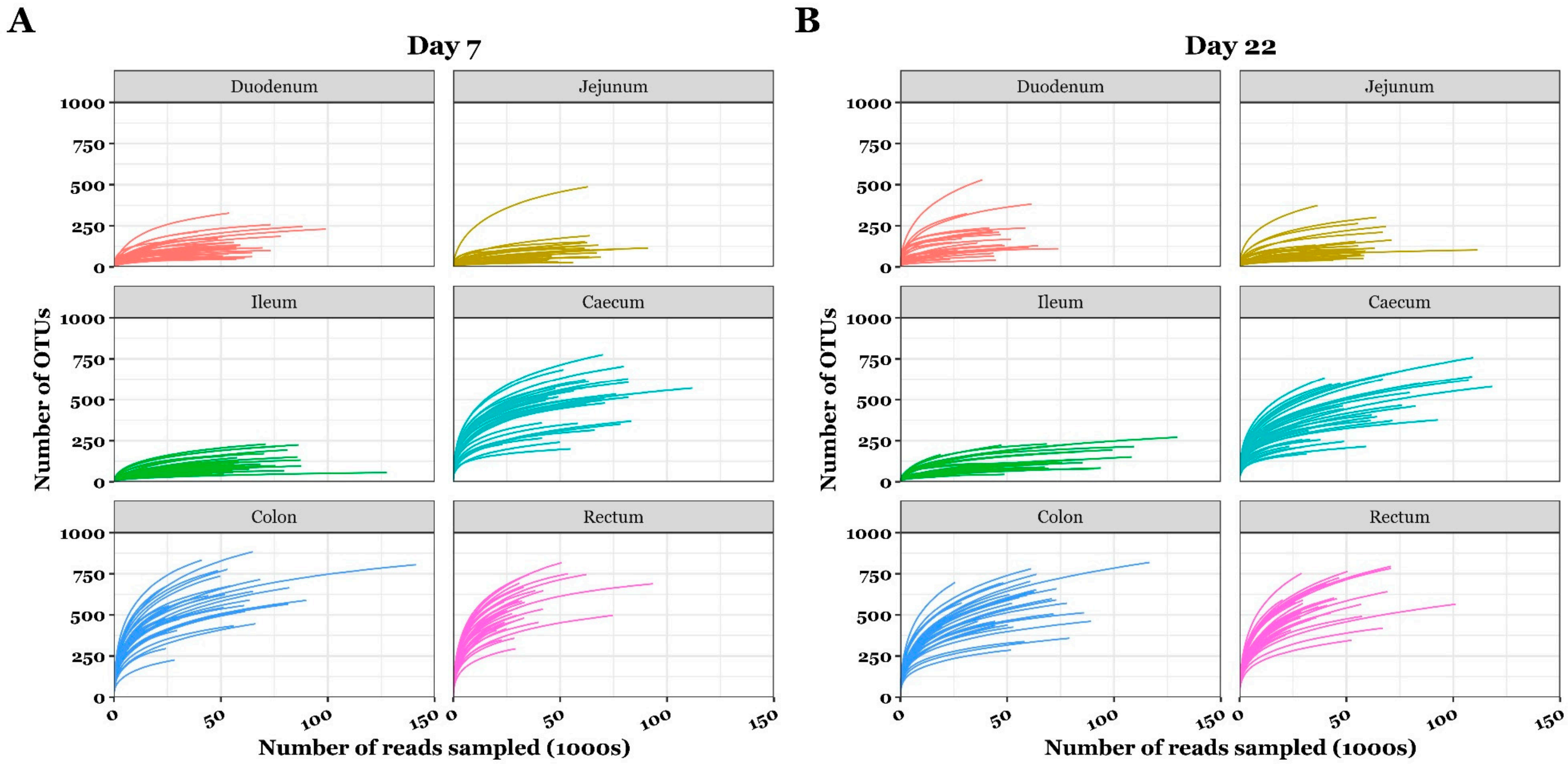

Figure S1. Rarefaction curves showing sequencing effort for all microbiota communities. Sample sizes for each day and GIT location are as follows: d7 – duodenum (*n* = 34), jejunum (*n* = 34), ileum (*n* = 36), caecum *n* = 36), colon (*n* = 32), rectum (*n* = 30); d22 – duodenum (*n* = 25), jejunum (*n* = 27), ileum (*n* = 35), caecum *n* = 36), colon (*n* = 34), rectum (*n* = 32).

Table S1. Alpha diversity of GIT microbial communities for control and prebiotic-fed pigs at day 7 and day 22 post-weaning. Mean (SEM).

GIT site	Day 7		Day 22		
	**CON**	**GXOS**	**CON**	**GXOS**	**XOS**
			ACE estimator		
Duodenum	137.62 (16.35)	146.87 (23.82)	215.03 (54.53)	179.26 (38.17)	164.64 (35.68)
Jejunum	115.1 (13.29)	150.15 (42.21)	133.76 (26.23)	209.87 (43.54)	122.07 (14.51)
Ileum	131.24 (13.8)	146.88 (17.28)	153.22 (15.29)	170.82 (20.98)	142.29 (22.32)
Caecum	481.53 (27.56)	547.8 (36.65)	445.3 (36.92)	509.97 (45.62)	426.47 (54.34)
Colon	593.66 (29.59)	659.91 (50.19)	583.58 (43.18)	642.32 (41.18)	623.16 (47.74)
Rectum	615.59 (25.63)	625.28 (50.66)	642.77 (27.94)	644.9 (48.21)	650.23 (40.74)
			Chao richness		
Duodenum	118.83 (15.23)	128.76 (17.01)	205.95 (52.1)	161.88 (37.12)	162.16 (37.05)
Jejunum	87.99 (8.94)	127.34 (34.92)	117.86 (23.3)	175.14 (38.02)	108.68 (10)
Ileum	103.98 (10.52)	120.9 (14.81)	121.64 (11.61)	137.12 (17.92)	105.75 (15.98)
Caecum	462.19 (25.67)	527.65 (35.28)	429.46 (31.31)	473.98 (41)	402.56 (46.77)
Colon	576.51 (29.53)	628.71 (47.07)	556.1 (39.52)	592.25 (41.2)	575.6 (37.86)
Rectum	603.83 (25.77)	610.88 (48.82)	625.73 (25.16)	620.74 (47.53)	616.26 (37.82)
			Shannon index		
Duodenum	1.28 (0.06)	1.6 (0.15)	2.02 (0.22)	1.71 (0.35)	1.74 (0.29)
Jejunum	1.12 (0.06)[a]	1.38 (0.13)[b]	1.2 (0.18)	1.19 (0.17)	1.33 (0.15)
Ileum	1.4 (0.06)	1.52 (0.15)	1.56 (0.14)	1.38 (0.12)	1.58 (0.11)
Caecum	3.68 (0.09)[a]	3.98 (0.07)[b]	3.7 (0.09)	3.73 (0.14)	3.55 (0.14)
Colon	3.94 (0.09)	4.18 (0.1)	3.87 (0.08)	3.82 (0.14)	3.78 (0.09)
Rectum	4.15 (0.09)	4.21 (0.1)	4 (0.06)	3.95 (0.11)	3.99 (0.09)
			Inverse Simpson diversity		
Duodenum	2.74 (0.16)	3.4 (0.5)	4.28 (0.58)	4.27 (1.28)	3.69 (0.88)
Jejunum	2.4 (0.14)	2.69 (0.26)	2.52 (0.36)	2.29 (0.24)	2.61 (0.32)
Ileum	3.14 (0.17)	3.39 (0.4)	3.58 (0.43)	3.05 (0.42)	3.55 (0.36)
Caecum	18.63 (1.5)[a]	24.26 (1.79)[b]	19.93 (1.93)	22 (2.68)	17.46 (2.71)
Colon	23.21 (2.34)	29.25 (3.28)	20.19 (2.54)	19.94 (2.63)	18.47 (2.32)
Rectum	28.67 (2.52)	29.37 (3.17)	20.63 (1.95)	20.35 (2.56)	22.73 (2.66)

For each timepoint, mean values within a row with a unique superscript letter differ significantly ($p < 0.05$). Sample sizes for each day, GIT location and diet group are as follows: d7 – duodenum (CON: $n = 23$, GXOS: $n = 11$), jejunum (CON: $n = 22$, GXOS: $n = 12$), ileum (CON: $n = 24$, GXOS: $n = 12$), caecum (CON: $n = 24$, GXOS: $n = 12$), colon (CON: $n = 21$, GXOS: $n = 11$), rectum (CON: $n = 20$, GXOS: $n = 10$); d22 – duodenum (CON: $n = 9$, GXOS: $n = 8$, XOS: $n = 8$), jejunum (CON: $n = 9$, GXOS: $n = 10$, XOS: $n = 8$), ileum (CON: $n = 12$, GXOS: $n = 12$, XOS: $n = 11$), caecum (CON: $n = 12$, GXOS: $n = 12$, XOS: $n = 12$), colon (CON: $n = 10$, GXOS: $n = 12$, XOS: $n = 12$), rectum (CON: $n = 10$, GXOS: $n = 10$, XOS: $n = 12$).

Table S2. Beta diversity showing significant differences in AMOVA between control and prebiotic-fed pigs at day 7 and day 22 post-weaning. Values shown are *p*-values generated from AMOVA analysis in Mothur.

GIT site	Day 7	Day 22		
	CON–GXOS	CON–GXOS	CON–XOS	GXOS–XOS
Yue & Clayton Dissimilarity				
Duodenum	0.262	0.314	0.667	0.223
Jejunum	0.422	0.398	0.441	0.717
Ileum	0.047	0.795	0.29	0.567
Caecum	0.224	0.685	0.676	0.898
Colon	0.359	0.923	0.985	0.969
Rectum	0.897	0.728	0.487	0.854
Bray-Curtis Dissimilarity				
Duodenum	0.131	0.606	0.71	0.332
Jejunum	0.201	0.624	0.44	0.792
Ileum	0.021	0.811	0.393	0.499
Caecum	0.138	0.731	0.691	0.899
Colon	0.317	0.803	0.889	0.952
Rectum	0.588	0.626	0.637	0.905
Jaccard Similarity				
Duodenum	0.066	0.677	0.847	0.802
Jejunum	0.091	0.467	0.568	0.39
Ileum	0.014	0.627	0.848	0.6
Caecum	0.156	0.635	0.389	0.816
Colon	0.576	0.733	0.854	0.986
Rectum	0.471	0.577	0.452	0.887

Sample sizes for each day, GIT location and diet group are as follows: d7 – duodenum (CON: $n = 23$, GXOS: $n = 11$), jejunum (CON: $n = 22$, GXOS: $n = 12$), ileum (CON: $n = 24$, GXOS: $n = 12$), caecum (CON: $n = 24$, GXOS: $n = 12$), colon (CON: $n = 21$, GXOS: $n = 11$), rectum (CON: $n = 20$, GXOS: $n = 10$); d22 – duodenum (CON: $n = 9$, GXOS: $n = 8$, XOS: $n = 8$), jejunum (CON: $n = 9$, GXOS: $n = 10$, XOS: $n = 8$), ileum (CON: $n = 12$, GXOS: $n = 12$, XOS: $n = 11$), caecum (CON: $n = 12$, GXOS: $n = 12$, XOS: $n = 12$), colon (CON: $n = 10$, GXOS: $n = 12$, XOS: $n = 12$), rectum (CON: $n = 10$, GXOS: $n = 10$, XOS: $n = 12$)

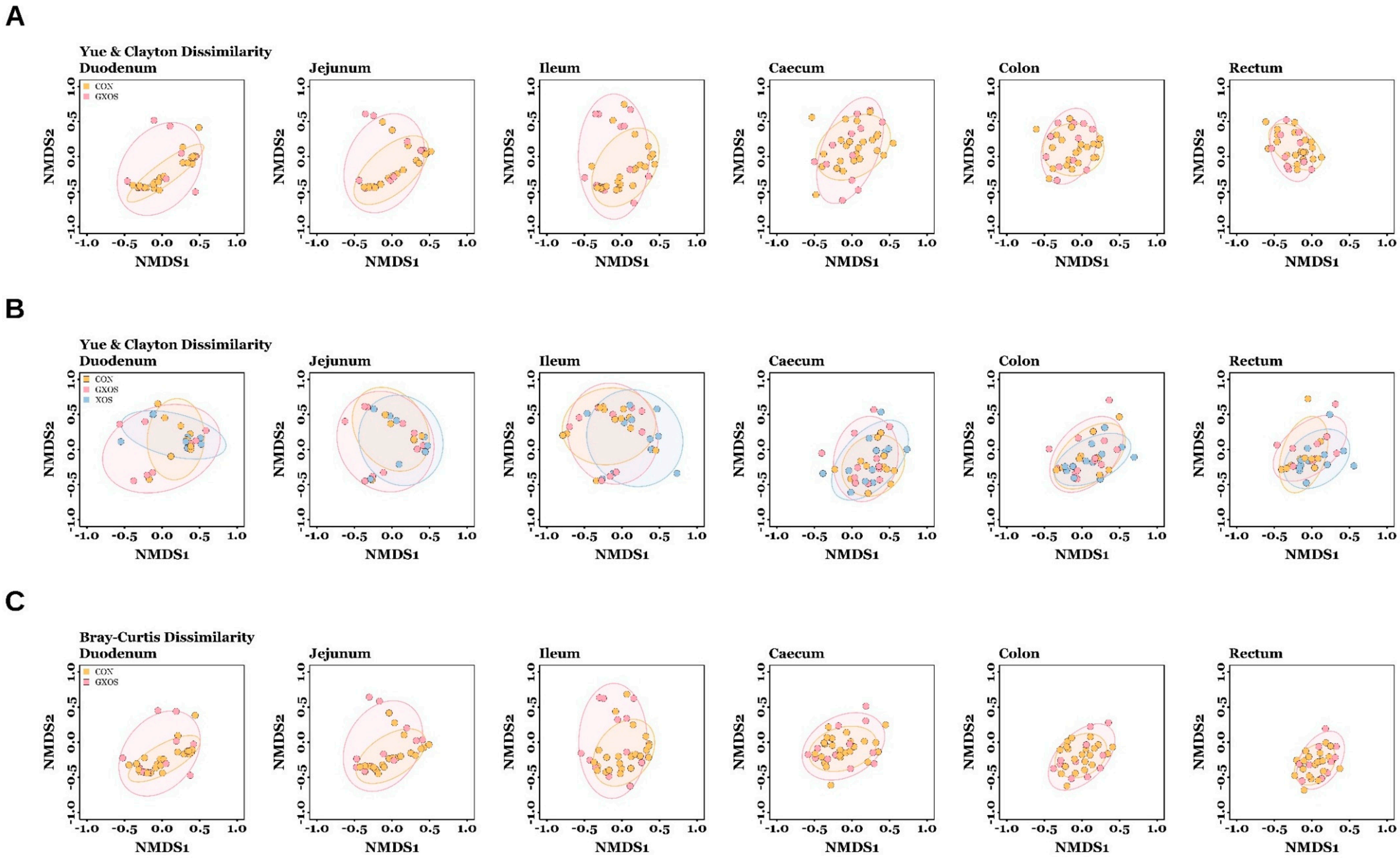
A
Yue & Clayton Dissimilarity
Duodenum
CON
GXOS
Jejunum
Ileum
Caecum
Colon
Rectum
NMDS2
NMDS1
B
Yue & Clayton Dissimilarity
Duodenum
CON
GXOS
XOS
Jejunum
Ileum
Caecum
Colon
Rectum
NMDS2
NMDS1
C
Bray-Curtis Dissimilarity
Duodenum
CON
GXOS
Jejunum
Ileum
Caecum
Colon
Rectum
NMDS2
NMDS1

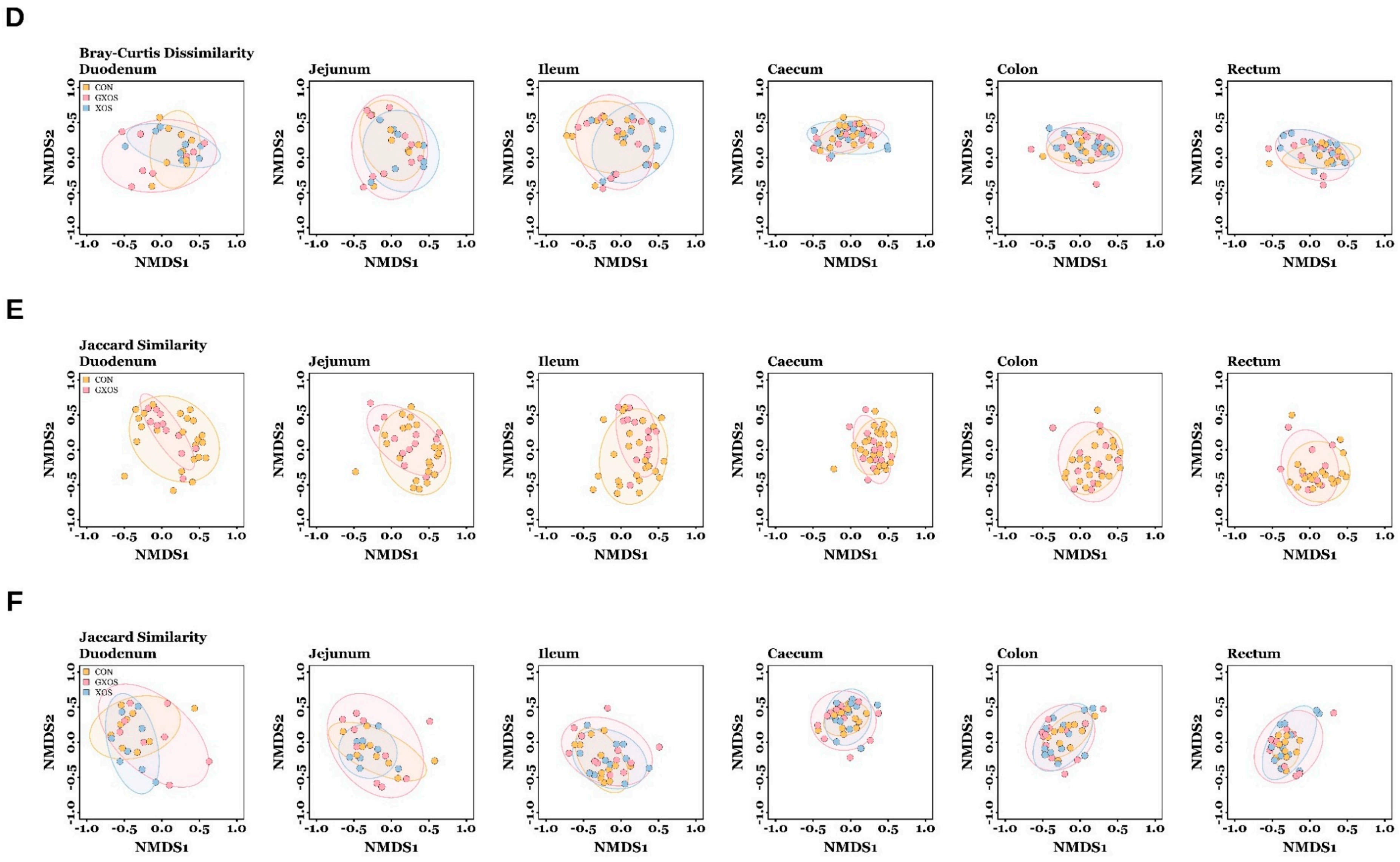

Figure S2. Supplementary NMDS plots showing β-diversity of GIT microbial communities for control and prebiotic-fed pigs at day 7 and day 22 post-weaning. Yue & Clayton Dissimilarity on d7 (A) and d22 (B). Bray Curtis Dissimilarity on d7 (C) and d22 (D). Jaccard Similarity on d7 (E) and d22 (F). Sample sizes for each day, GIT location and diet group are as follows: d7 – duodenum (CON: *n* = 23, GXOS: *n* = 11), jejunum (CON: *n* = 22, GXOS: *n* = 12), ileum (CON: *n* = 24, GXOS: *n* = 12), caecum (CON: *n* = 24, GXOS: *n* = 12), colon (CON: *n* = 21, GXOS: *n* = 11), rectum (CON: *n* = 20, GXOS: *n* = 10); d22 – duodenum (CON: *n* = 9, GXOS: *n* = 8, XOS: *n* = 8), jejunum (CON: *n* = 9, GXOS: *n* = 10, XOS: *n* = 8), ileum (CON: *n* = 12, GXOS: *n* = 12, XOS: *n* = 11), caecum (CON: *n* = 12, GXOS: *n* = 12, XOS: *n* = 12), colon (CON: *n* = 10, GXOS: *n* = 12, XOS: *n* = 12), rectum (CON: *n* = 10, GXOS: *n* = 10, XOS: *n* = 12).

Table S3. Histomorphological measurements of control and prebiotic-supplemented pigs at day 7 and day 22 post-weaning.

	Day 7				Day 22				
	Diet		Pooled SEM	p-value[1]	Diet			Pooled SEM	p-value[2]
Jejunum	CON	GXOS			CON	GXOS	XOS		
Villus height (µm)	317	316	10.70	0.903	318	318	318	14.20	0.981
Crypt depth (µm)	240	243	5.85	0.631	321	305	330	6.60	0.317
Villus GC per mm^2	540	435	20.95	0.028	474	551	501	26.18	0.529
Crypt GC per mm^2	2858	2720	64.77	0.243	3243	3484	3294	99.85	0.716
VCR	1.43	1.53	0.07	0.987	1.06	1.13	1.12	0.06	0.843
Ileum									
Villus height (µm)	241	244	8.63	0.830	270	283	263	9.45	0.933
Crypt depth (µm)	244	232	5.62	0.326	280	292	297	5.53	0.478
Villus GC per mm^2	586	618	31.70	0.534	620	640	541	36.15	0.355
Crypt GC per mm^2	3900	3766	109.14	0.657	4102	4287	4190	99.69	0.607
VCR	1.12	1.02	0.05	0.406	1.04	0.99	0.91	0.04	0.552

[1] p-values from Wilcoxon rank-sum tests; [2] p-values from Kruskal-Wallis tests. Values are means of the average measurement per pig, averaged across 12 replicate pigs per diet group. GC, goblet cells; VCR, villus-to-crypt ratio.

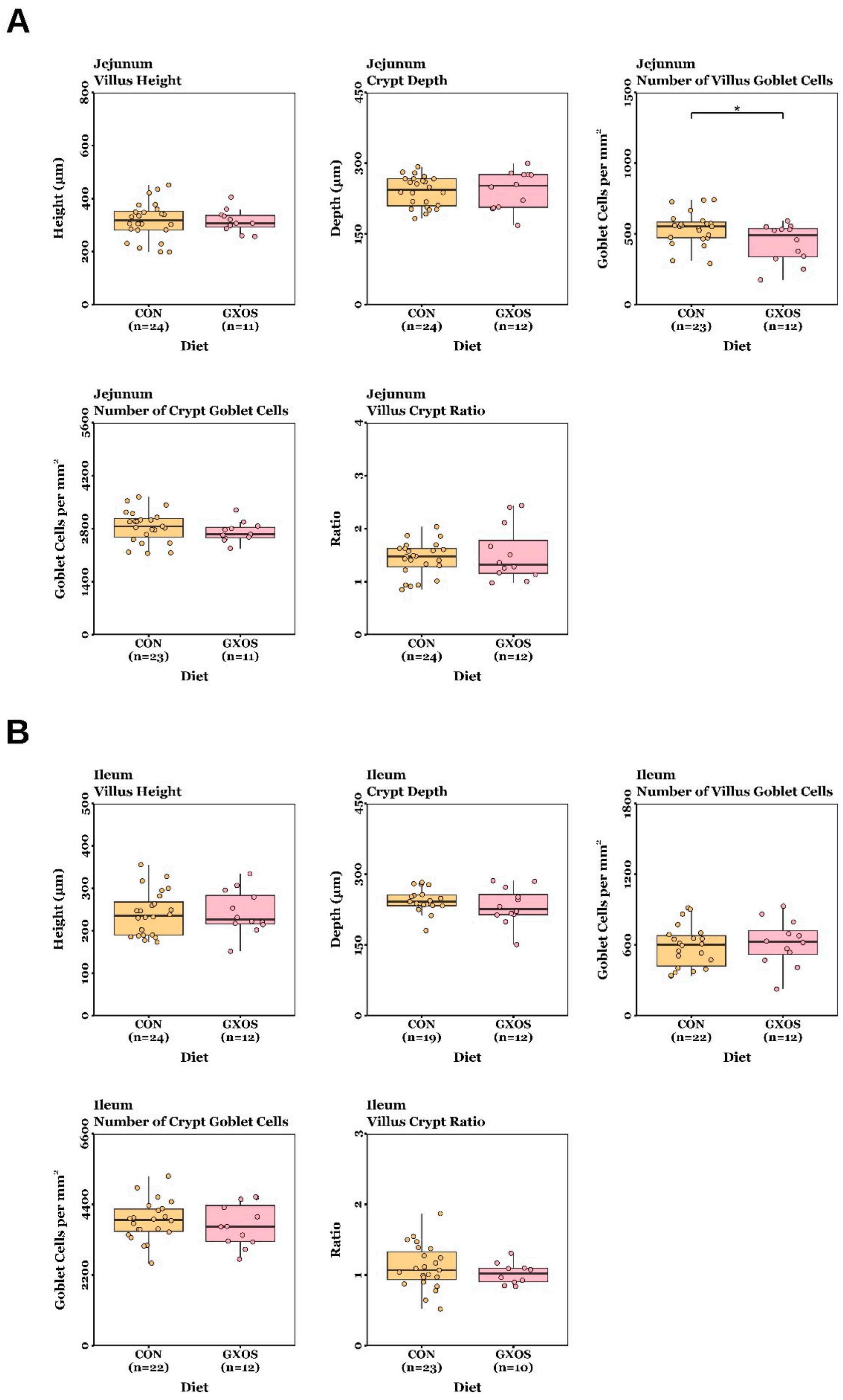
A
Jejunum
Villus Height
Height (µm)
CON (n=24)
GXOS (n=11)
Diet
Jejunum
Crypt Depth
Depth (µm)
CON (n=24)
GXOS (n=12)
Diet
Jejunum
Number of Villus Goblet Cells
Goblet Cells per mm²
*
CON (n=23)
GXOS (n=12)
Diet
Jejunum
Number of Crypt Goblet Cells
Goblet Cells per mm²
CON (n=23)
GXOS (n=11)
Diet
Jejunum
Villus Crypt Ratio
Ratio
CON (n=24)
GXOS (n=12)
Diet
B
Ileum
Villus Height
Height (µm)
CON (n=24)
GXOS (n=12)
Diet
Ileum
Crypt Depth
Depth (µm)
CON (n=19)
GXOS (n=12)
Diet
Ileum
Number of Villus Goblet Cells
Goblet Cells per mm²
CON (n=22)
GXOS (n=12)
Diet
Ileum
Number of Crypt Goblet Cells
Goblet Cells per mm²
CON (n=22)
GXOS (n=12)
Diet
Ileum
Villus Crypt Ratio
Ratio
CON (n=23)
GXOS (n=10)
Diet

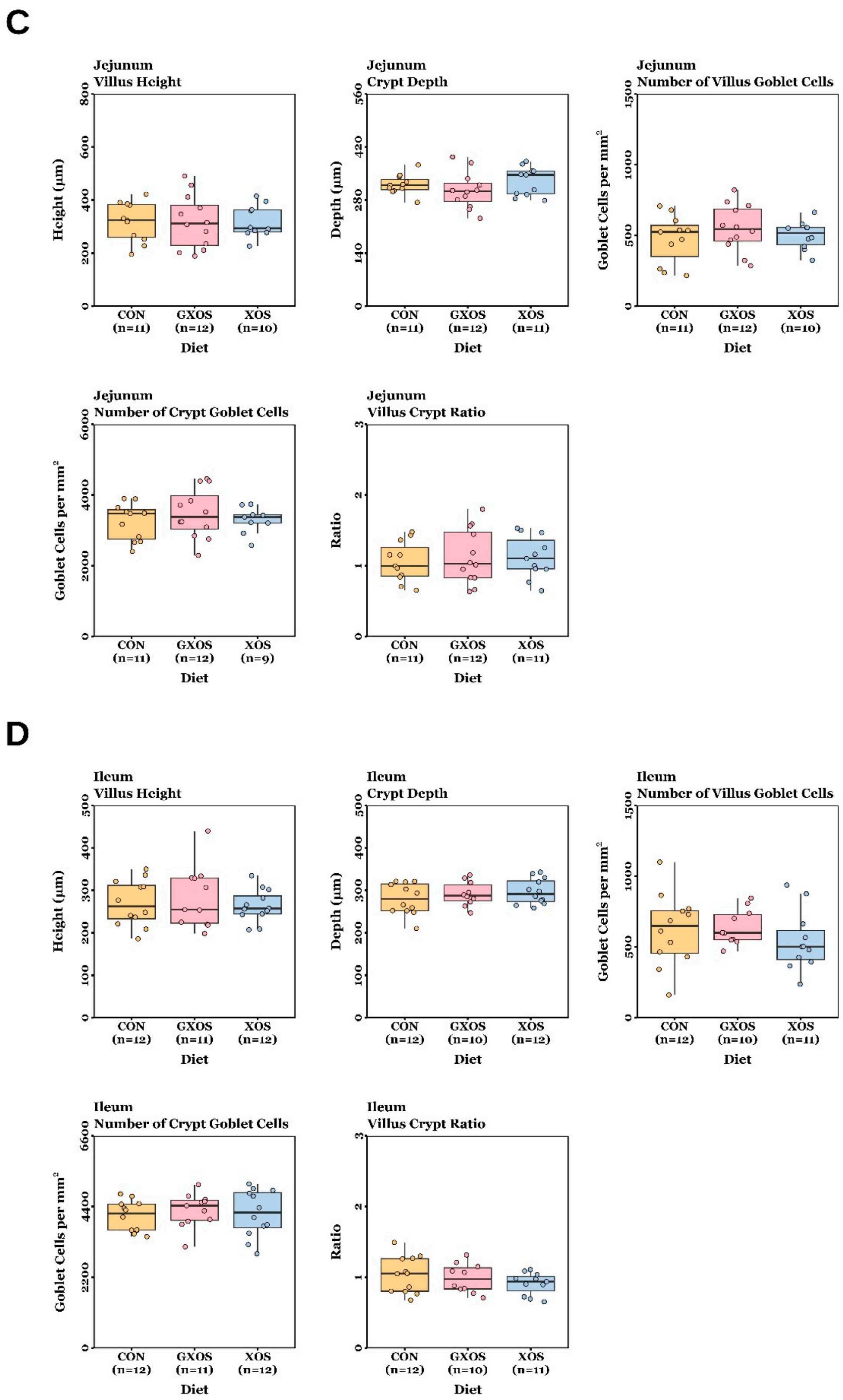

Figure S3. Complementary boxplots showing histomorphological measurements of control and prebiotic-supplemented pigs at day 7 (A & B) and day 22 (C & D) post-weaning. Wilcoxon rank sum test; *, $p < 0.001$. Boxplots are presented as median, Q1 and Q3, while whiskers extend from the smallest value $\geqslant$ Q1 – 1.5 $\times$ IQR up to the greatest value $\leqslant$ Q3 – 1.5 $\times$ IQR.

Table S4. Feed formulations of CON, GXOS, and XOS diets used throughout the study. Feed formulations for starter (day 1 – day 7 post-weaning; d1 – d7), link (d8 – d22) and grower (d23 – d54) phases.

Feed component inclusion (%)	Starter phase			Link phase			Grower phase		
	CON	GXOS	XOS	CON	GXOS	XOS	CON	GXOS	XOS
Micronised barley	10.00	10.00	10.00	15.00	15.00	15.00	0.00	0.00	0.00
Barley	0.00	0.00	0.00	0.00	0.00	0.00	15.00	15.00	15.00
Wheat (raw whole meal)	20.44	20.44	20.39	37.91	37.91	37.86	47.87	47.86	47.82
Micronised wheat (meal)	10.00	10.00	10.00	5.00	5.00	5.00	0.00	0.00	0.00
Wheatfeed	0.00	0.00	0.00	0.00	0.00	0.00	1.03	1.03	1.03
Micronised oats	10.00	10.00	10.00	0.00	0.00	0.00	0.00	0.00	0.00
Fishmeal	7.25	7.25	7.25	5.77	5.77	5.77	0.00	0.00	0.00
Hypro soya bean meal	19.00	19.00	19.00	24.00	24.00	24.00	27.10	27.10	27.10
Premix 1[1]	0.50	0.50	0.50	0.50	0.50	0.50	0.00	0.00	0.00
Dried skim milk	4.00	4.00	4.00	0.00	0.00	0.00	0.00	0.00	0.00
Whey powder	11.41	11.41	11.41	7.25	7.25	7.25	3.62	3.62	3.62
L-lysine HCl	0.312	0.312	0.312	0.236	0.236	0.236	0.483	0.483	0.483
L-methionine	0.19	0.19	0.19	0.124	0.124	0.124	0.189	0.189	0.189
L-threonine	0.19	0.19	0.19	0.121	0.121	0.121	0.215	0.215	0.215
L-tryptophan	0.029	0.029	0.029	0.00	0.00	0.00	0.009	0.009	0.009
L-valine	0.066	0.066	0.066	0.00	0.00	0.00	0.10	0.10	0.10
**GOS**	**0.00**	**0.15**	**0.00**	**0.00**	**0.00**	**0.00**	**0.00**	**0.00**	**0.00**
**XOS**	**0.00**	**0.00**	**0.00**	**0.00**	**0.05**	**0.05**	**0.00**	**0.05**	**0.05**
Vitamin E	0.02	0.02	0.02	0.01	0.01	0.01	0.03	0.03	0.03
Sucram	0.01	0.01	0.01	0.01	0.01	0.01	0.01	0.01	0.01
Dicalcium phosphate	0.84	0.84	0.84	0.97	0.97	0.97	1.95	1.95	1.95
Soya oil	5.74	5.74	5.74	2.94	2.94	2.94	1.68	1.68	1.68
Pure dried vacuum salt	0.00	0.00	0.00	0.16	0.16	0.16	0.41	0.41	0.41
Premix 2[2]	0.00	0.00	0.00	0.00	0.00	0.00	0.25	0.25	0.25
Copper sulphate	0.00	0.00	0.00	0.00	0.00	0.00	0.03	0.03	0.03
Iron (II) chelate of glycine (B-traxim 2c Fe-220; M60-5000)	0.00	0.00	0.00	0.00	0.00	0.00	0.03	0.03	0.03

[1]Premix provides per kg of feed: 13750 IU Vitamin A; 2100 IU Vitamin D3; 150 mg Vitamin E; 6.0 mg Vitamin K3 – Menadione; 1.6 mg Vitamin B1; 6.0 mg Riboflavin; 2.3 mg Vitamin B6; 0.028 mg Vitamin B12; 25.0 mg Niacin; 13.3 mg Pantothenic acid; 1.0 mg Folic acid; 0.150 mg Biotin; 1.0 mg Iodine (calcium iodate, anhydrous); 0.25 mg Selenium (Sodium selenite); 150 mg Iron (Iron (II) sulphate monohydrate); 140 mg Copper (Copper (II) sulphate pentahydrate); 110 mg Zinc (Zinc sulphate monohydrate); 40 mg Manganese (Manganous sulphate monohydrate).

[2]Premix provides per kg of feed: 10500 IU Vitamin A; 2250 IU Vitamin D3; 50.0 mg Vitamin E; 4.0 mg Vitamin K; 0.874 mg Vitamin K3 – Menadione; 1.5 mg Vitamin B1; 4.0 mg Riboflavin; 3.5 mg Vitamin B6; 0.015 mg Vitamin B12; 20.0 mg Niacin; 12.0 mg Calcium pantothenate; 11.035 mg Pantothenic acid; 2.0 mg Folic acid; 0.2 mg Biotin; 1.0 mg Iodine (calcium iodate, anhydrous); 0.25 mg Selenium (Sodium selenite); 80.0 mg Iron (Iron (II) sulphate monohydrate); 15.0 mg Copper (Copper (II) sulphate pentahydrate); 100 mg Zinc (Zinc sulphate monohydrate); 50.0 mg Manganese (Manganous sulphate monohydrate); 10.0 mg Citric acid.

Table S5. Nutritional composition of creep feed offered to all piglets from 15 to 28 days of age.

Constituent	Amount/kg
Crude protein (g)	240
Crude fibre (g)	26
Crude oil and fats (g)	130
Crude ash (g)	70
Lysine (g)	16
Methionine (g)	3
Calcium (g)	6
Sodium (g)	3.5
Phosphorus (g)	6
Vitamin A (iu)	12,500
Vitamin D3 (iu)	2,000
Vitamin E (iu)	95

Table S6. Nutritional composition of supplementary milk offered to all piglets from birth to 28 days of age.

Constituent	Inclusion (%)
Crude protein	20
Crude fibre	0.1
Crude oil and fats	14
Crude ash	6.1
Lysine	1.8
Methionine	0.7
Calcium	0.55
Sodium	0.47
Phosphorus	0.5

Table S7. Assay IDs for target and reference genes included in the Qiagen Custom RT^2 Profiler PCR Array used for quantitative gene expression analysis.

Full gene name	Abbreviation	GenBank accession	Qiagen assay ID
**Occludin**	OCLN	NM_001163647	PPS00567A
**Zonula occludens-1**	ZO-1	XM_003480423	PPS71986A
**Zonula occludens-2**	ZO-2	NM_001206404	PPS05196A
**Claudin-2**	CLDN-2	NM_001161638	PPS01521A
**Claudin-3**	CLDN-3	NM_001160075	PPS01171A
**Interleukin-1β**	IL-1β	NM_214055	PPS00461A
**Interleukin-6**	IL-6	NM_214399	PPS00991A
**Interleukin-8**	IL-8	NM_213867	PPS00237A
**Interleukin-10**	IL-10	NM_214041	PPS00445A
**Intestinal alkaline phosphatase**	ALPI	XM_003133729	PPS71602A
**Ribosomal protein L4**	RPL4	XM_005659862	PPS07992A
**Glyceraldehyde-3-phosphate dehydrogenase**	GAPDH	NM_001206359	PPS00192A
**Pig genomic DNA contamination**	SGDC	SA_00133	PPS71596A
**Positive PCR control**	PPC	SA_00103	PPX63339A
**Reverse transcription control**	RTC	SA_00104	PPX63340A

Table S8. The mean relative abundance of bacterial taxa from the GIT digesta of piglets fed a control or prebiotic-supplemented diet on day 7 post-weaning. Values are mean relative abundance (SEM).

Taxon	Diet and GIT section											
	Duodenum		Jejunum		Ileum		Caecum		Colon		Rectum	
	CON	GXOS	CON	GXOS	CON	GXOS	CON	GXOS	CON	GXOS	CON	GXOS
Lactobacillus	60.83	52.38	66.5	53.81	53.38	36.93	13.31	10.65	9.36	6.32	5.99	4.63
	(2.57)	(5.37)	(3.05)	(6.75)	(3.55)	(6.62)	(1.12)	(1.73)	(0.79)	(0.85)	(0.54)	(0.78)
Limosilactobacillus	28.49	20.06	23.57	16.61	24.08	12.53	5.12	4.02	4.55	4.02	4.33	4.01
	(2.33)	(3.73)	(2.03)	(3.22)	(1.88)	(3.00)	(0.43)	(0.63)	(0.37)	(0.62)	(0.31)	(0.53)
Sarcina	1.13	9.13	5.79	16.52	5.62	23.94	0.17	1.15	0.15	0.15	0.01	0.08
	(0.78)	(5.34)	(3.39)	(8.91)	(2.80)	(9.56)	(0.09)	(0.62)	(0.08)	(0.07)	(<0.01)	(0.02)
Megasphaera	0.18	2.42	0.05	0.76	0.03	0.93	10.19	5.92	9.94	6.07	7.92	5.68
	(0.06)	(2.18)	(0.02)	(0.61)	(0.01)	(0.91)	(1.45)	(1.23)	(1.38)	(1.17)	(1.08)	(0.92)
Phascolarctobacterium	0.04	0.11	0.05	0.06	0.01	0.01	4.53	3.68	4.67	4.69	4.92	4.52
	(0.01)	(0.05)	(0.03)	(0.04)	(<0.01)	(<0.01)	(0.47)	(0.57)	(0.42)	(0.62)	(0.34)	(0.71)
Ligilactobacillus	2.42	2.93	1.18	5.78	3.39	5.18	0.65	0.93	0.24	0.31	0.11	0.21
	(1.28)	(1.3)	(0.44)	(3.06)	(1.00)	(2.17)	(0.19)	(0.40)	(0.07)	(0.14)	(0.04)	(0.08)
Oscillospiraceae UC	0.04	0.04	0.02	0.05	0.02	0.06	1.61	2.07	2.39	3.26	3.33	3.30
	(0.01)	(0.02)	(0.01)	(0.02)	(0.01)	(0.02)	(0.14)	(0.36)	(0.21)	(0.51)	(0.37)	(0.56)
Blautia	0.11	0.14	0.04	0.08	0.06	0.03	2.93	3.48	2.33	2.44	1.75	2.12
	(0.03)	(0.05)	(0.01)	(0.03)	(0.02)	(0.01)	(0.36)	(0.82)	(0.28)	(0.63)	(0.23)	(0.36)
Lachnospiraceae UC	0.05	0.05	0.03	0.04	0.01	0.02	1.87	2.12	2.42	2.54	2.88	2.81
	(0.02)	(0.02)	(0.02)	(0.02)	(<0.01)	(0.01)	(0.22)	(0.31)	(0.25)	(0.39)	(0.34)	(0.42)
Selenomonadaceae UC	0.01	0.01	0.01	0.04	0.01	0.01	3.16	2.55	2.88	1.44	2.34	2.33
	(<0.01)	(<0.01)	(0.01)	(0.03)	(<0.01)	(<0.01)	(0.73)	(0.80)	(0.56)	(0.36)	(0.44)	(0.5)
Other Bacillota	2.74	6.62	0.89	3.91	2.87	3.87	14.32	17.30	14.06	16.06	12.60	15.19
	(1.44)	(1.96)	(0.26)	(1.22)	(0.92)	(1.01)	(1.1)	(1.92)	(0.64)	(2.09)	(0.75)	(1.30)
Segatella	0.05	0.04	0.06	0.17	0.08	0.04	13.81	10.99	15.37	13.96	13.75	14.83
	(0.01)	(0.01)	(0.04)	(0.11)	(0.04)	(0.03)	(1.34)	(1.50)	(1.45)	(2.08)	(1.47)	(2.44)
Prevotellaceae UC	0.07	0.16	0.06	0.14	0.06	0.09	7.38	6.70	9.28	9.45	11.43	10.38
	(0.03)	(0.08)	(0.03)	(0.08)	(0.02)	(0.04)	(1.42)	(1.39)	(1.22)	(1.08)	(1.07)	(1.11)
Prevotellamassilia	0.02	0.01	0.03	0.09	0.01	0.01	3.79	4.73	3.16	4.16	3.08	4.15
	(0.01)	(<0.01)	(0.02)	(0.04)	(0.01)	(0.01)	(0.57)	(0.51)	(0.44)	(0.67)	(0.43)	(0.91)
Bacteroidales UC	0.01	0.01	0.03	0.03	0.01	0.01	2.48	2.08	2.97	2.93	4.03	2.81
	(<0.01)	(<0.01)	(0.03)	(0.02)	(<0.01)	(<0.01)	(0.44)	(0.37)	(0.47)	(0.42)	(0.40)	(0.46)

Other Bacteroidota	0.08 (0.02)	0.07 (0.04)	0.05 (0.03)	0.09 (0.06)	0.03 (0.01)	0.02 (0.01)	4.74 (0.42)	5.12 (0.72)	7.2 (0.55)	7.77 (0.79)	9.96 (0.91)	9.17 (1.13)
Escherichia/Shigella	2.40 (1.35)	2.65 (2.46)	0.63 (0.38)	0.15 (0.04)	8.47 (2.83)	8.67 (3.98)	2.96 (1.15)	2.70 (1.04)	1.20 (0.65)	0.80 (0.26)	0.42 (0.16)	0.58 (0.17)
Other Pseudomonadota	0.94 (0.34)	1.71 (0.73)	0.71 (0.38)	0.82 (0.29)	1.38 (0.52)	6.82 (3.05)	1.65 (0.31)	3.96 (0.91)	0.93 (0.11)	1.23 (0.23)	1.24 (0.31)	1.12 (0.20)
Treponema	0.01 (<0.01)	0.00 (<0.01)	0.01 (0.01)	0.02 (0.02)	0.00 (<0.01)	0.00 (<0.01)	1.60 (0.44)	2.00 (0.63)	2.89 (0.54)	4.84 (1.23)	4.06 (0.63)	4.58 (1.45)
Others	0.02 (0.01)	0.08 (0.03)	0.01 (<0.01)	0.04 (0.02)	0.03 (0.01)	0.05 (0.01)	0.18 (0.03)	0.39 (0.07)	0.18 (0.03)	0.34 (0.07)	0.24 (0.04)	0.32 (0.06)

UC = unclassified.

Table S9. The mean relative abundance of bacterial taxa from the GIT digesta of piglets fed a control or prebiotic-supplemented diet on day 22 post-weaning. Values are mean relative abundance (SEM).

Taxon	Diet and GIT section																	
	Duodenum			Jejunum			Ileum			Caecum			Colon			Rectum		
	CON	GXOS	XOS	CON	GXOS	XOS	CON	GXOS	XOS	CON	GXOS	XOS	CON	GXOS	XOS	CON	GXOS	XOS
Lactobacillus	45.33	54.4	48.79	37.72	48.41	55.26	26.22	33.08	38.40	9.28	11.16	11.84	8.84	10.64	8.44	5.35	9.31	6.68
	(6.57)	(10.86)	(8.75)	(7.48)	(10.4)	(9.18)	(6.69)	(7.47)	(7.48)	(1.58)	(2.89)	(3.00)	(1.75)	(2.87)	(1.88)	(1.16)	(2.12)	(1.29)
Sarcina	13.47	8.81	19.29	42.65	27.33	22.12	32.87	28.58	14.10	3.76	2.24	1.87	1.46	0.88	1.21	0.83	0.55	0.78
	(4.08)	(3.48)	(8.40)	(11.11)	(11.1)	(9.96)	(8.19)	(8.91)	(5.07)	(1.31)	(0.7)	(0.65)	(0.4)	(0.2)	(0.35)	(0.2)	(0.19)	(0.18)
Limosilactobacillus	19.81	12.5	13.88	15.00	12.33	14.92	8.59	13.19	12.27	3.00	3.56	3.88	3.63	3.32	3.46	2.29	3.52	3.14
	(4.50)	(2.68)	(3.85)	(3.75)	(2.93)	(3.06)	(2.00)	(3.55)	(2.81)	(0.56)	(1.00)	(1.45)	(0.60)	(0.81)	(1.04)	(0.5)	(0.68)	(0.62)
Megasphaera	1.25	1.67	4.17	0.16	0.26	1.00	0.05	0.11	0.17	11.51	10.06	11.67	9.95	10.87	12.78	8.98	10.25	10.56
	(0.64)	(1.32)	(3.33)	(0.09)	(0.15)	(0.55)	(0.01)	(0.05)	(0.08)	(1.85)	(1.50)	(2.81)	(1.77)	(2.26)	(2.66)	(1.66)	(2.11)	(1.71)
Clostridium sensu	3.76	0.77	0.68	0.30	0.23	0.79	7.87	7.58	12.24	0.55	1.12	0.83	0.47	0.93	0.83	0.55	0.75	0.87
stricto	(3.05)	(0.4)	(0.26)	(0.26)	(0.09)	(0.66)	(3.26)	(4.33)	(4.14)	(0.21)	(0.46)	(0.22)	(0.19)	(0.35)	(0.31)	(0.22)	(0.30)	(0.31)
Blautia	1.32	1.40	1.29	0.42	0.30	0.37	0.17	0.11	0.08	4.95	4.01	3.93	3.73	3.60	3.28	2.92	3.31	3.14
	(0.72)	(1.02)	(0.89)	(0.26)	(0.1)	(0.14)	(0.07)	(0.03)	(0.03)	(0.88)	(0.43)	(0.62)	(0.45)	(0.45)	(0.36)	(0.29)	(0.52)	(0.36)
Butyricicoccus	0.38	0.27	0.18	0.09	0.16	0.11	0.04	0.06	0.03	3.46	3.71	2.93	2.66	3.16	2.55	2.34	3.10	2.73
	(0.15)	(0.16)	(0.11)	(0.06)	(0.08)	(0.07)	(0.02)	(0.04)	(0.01)	(0.41)	(0.58)	(0.48)	(0.29)	(0.46)	(0.25)	(0.26)	(0.53)	(0.32)
Oscillospiraceae UC	0.32	0.39	0.22	0.09	0.09	0.17	0.03	0.03	0.02	2.15	1.99	1.97	2.63	2.98	2.55	3.01	3.43	3.15
	(0.13)	(0.30)	(0.12)	(0.06)	(0.05)	(0.1)	(0.01)	(0.01)	(0.01)	(0.34)	(0.33)	(0.36)	(0.29)	(0.35)	(0.29)	(0.31)	(0.35)	(0.46)
Streptococcus	0.97	2.44	1.17	0.55	0.52	0.65	5.32	1.53	7.07	0.56	0.39	0.38	0.43	0.2	0.36	0.3	0.16	0.40
	(0.33)	(1.54)	(0.38)	(0.21)	(0.22)	(0.23)	(2.79)	(0.6)	(5.21)	(0.24)	(0.14)	(0.17)	(0.21)	(0.08)	(0.13)	(0.1)	(0.06)	(0.13)
Faecalibacterium	0.43	0.24	0.22	0.03	0.08	0.04	0.01	0.01	0.01	2.68	2.51	2.42	2.26	2.04	2.10	2.01	1.70	2.02
	(0.17)	(0.15)	(0.11)	(0.02)	(0.05)	(0.02)	(0.01)	(<0.01)	(0.01)	(0.37)	(0.28)	(0.35)	(0.44)	(0.31)	(0.34)	(0.25)	(0.27)	(0.26)
Selenomonadaceae	0.36	0.14	0.18	0.01	0.06	0.17	0.01	0.01	0.06	2.49	2.39	2.98	1.28	1.52	1.64	2.69	1.80	2.28
UC	(0.24)	(0.12)	(0.11)	(0.01)	(0.05)	(0.13)	(0.01)	(<0.01)	(0.04)	(1.00)	(0.59)	(1.17)	(0.40)	(0.36)	(0.35)	(0.47)	(0.41)	(0.51)
Lachnospiraceae UC	0.30	0.18	0.16	0.04	0.06	0.04	0.02	0.02	0.02	1.58	1.46	1.38	2.43	2.03	1.65	2.92	2.53	2.24
	(0.09)	(0.1)	(0.06)	(0.03)	(0.03)	(0.02)	(0.01)	(0.01)	(0.01)	(0.27)	(0.2)	(0.22)	(0.41)	(0.18)	(0.2)	(0.33)	(0.20)	(0.24)
Other Bacillota	5.92	5.01	4.69	2.23	7.71	2.76	5.02	3.90	5.68	17.72	19.27	17.88	17.4	17.96	18.53	17.01	17.60	18.05
	(1.71)	(2.57)	(1.67)	(0.86)	(5.66)	(1.2)	(1.38)	(1.03)	(2.00)	(1.34)	(1.69)	(1.59)	(1.19)	(1.55)	(1.71)	(1.27)	(1.53)	(1.62)
Segatella	1.74	0.54	0.75	0.03	0.24	0.09	0.12	0.14	0.05	9.28	10.65	13.75	15.76	14.62	17.01	18.78	15.57	17.10
	(0.86)	(0.28)	(0.44)	(0.01)	(0.18)	(0.03)	(0.08)	(0.07)	(0.02)	(1.8)	(1.93)	(3.00)	(2.78)	(2.42)	(2.73)	(2.47)	(2.40)	(2.52)
Prevotellaceae UC	0.58	0.46	0.36	0.03	0.15	0.31	0.06	0.04	0.1	4.26	4.39	4.54	6.82	6.45	5.15	7.37	6.38	6.13
	(0.28)	(0.27)	(0.12)	(0.02)	(0.12)	(0.27)	(0.03)	(0.02)	(0.07)	(0.63)	(0.86)	(0.92)	(0.91)	(1.38)	(0.75)	(0.73)	(1.28)	(0.92)
Prevotellamassilia	0.28	0.03	0.12	0.01	0.11	0.11	0.03	0.01	0.02	7.28	5.49	5.35	7.04	3.96	4.61	3.92	2.68	2.69
	(0.17)	(0.02)	(0.09)	(<0.01)	(0.1)	(0.07)	(0.02)	(<0.01)	(0.01)	(1.51)	(0.93)	(0.51)	(2.37)	(0.89)	(0.91)	(0.70)	(0.59)	(0.55)
Other Bacteroidota	0.58	0.36	0.29	0.01	0.12	0.05	0.06	0.04	0.04	4.84	6.15	5.54	6.61	7.18	7.39	8.74	7.22	8.08
	(0.23)	(0.18)	(0.10)	(<0.01)	(0.1)	(0.03)	(0.04)	(0.02)	(0.02)	(0.68)	(1.18)	(0.99)	(0.8)	(1.17)	(0.85)	(0.88)	(1.20)	(0.83)
Escherichia/Shigella	0.22	5.54	1.12	0.07	0.85	0.29	4.15	5.16	7.70	1.37	1.49	0.7	0.86	0.51	0.40	0.46	0.17	0.18
	(0.08)	(5.37)	(0.65)	(0.05)	(0.69)	(0.11)	(2.09)	(2.72)	(3.53)	(0.52)	(0.58)	(0.27)	(0.25)	(0.24)	(0.15)	(0.14)	(0.08)	(0.05)

Other Pseudomonadota	2.12 (1.26)	1.50 (0.78)	1.36 (0.57)	0.17 (0.11)	0.33 (0.15)	0.17 (0.07)	6.79 (3.39)	1.78 (1.65)	1.63 (0.69)	2.23 (0.57)	2.17 (0.52)	1.41 (0.36)	1.08 (0.22)	1.59 (0.52)	1.08 (0.25)	1.95 (1.37)	2.81 (1.27)	1.25 (0.42)
Others	0.04 (0.01)	0.17 (0.11)	0.05 (0.01)	0.02 (0.01)	0.03 (0.01)	0.03 (0.01)	0.13 (0.07)	0.23 (0.21)	0.02 (<0.01)	0.33 (0.07)	0.27 (0.06)	0.22 (0.04)	0.16 (0.03)	0.16 (0.04)	0.18 (0.04)	0.21 (0.05)	0.18 (0.04)	0.24 (0.05)

UC = unclassified.
